# A Novel Pyrazolopyrimidine Ligand of Human PGK1 and Stress Sensor DJ1 Modulates the Shelterin Complex and Telomere Length Regulation

**DOI:** 10.1016/j.neo.2019.07.008

**Published:** 2019-08-08

**Authors:** Alan E. Bilsland, Yu Liu, Andrew Turnbull, David Sumpton, Katrina Stevenson, Claire J. Cairney, Susan M. Boyd, Jon Roffey, David Jenkinson, W. Nicol Keith

**Affiliations:** * Institute of Cancer Sciences, University of Glasgow, Wolfson Wohl Cancer Research Centre, Garscube Estate, Switchback Road, Bearsden, Glasgow, G61 1QH, UK; † Cancer Research Technology Ltd., Wolfson Institute for Biomedical Research, The Cruciform Building, Gower Street, London, WC1E 6BT, UK; ‡ Cancer Research UK Beatson Institute, Garscube Estate, Switchback Road, Bearsden, Glasgow, G61 1BD, UK; § CompChem Solutions Ltd, St John's Innovation Centre, Cambridge, CB4 0WS, UK

## Abstract

Telomere signaling and metabolic dysfunction are hallmarks of cell aging. New agents targeting these processes might provide therapeutic opportunities, including chemoprevention strategies against cancer predisposition. We report identification and characterization of a pyrazolopyrimidine compound series identified from screens focused on cell immortality and whose targets are glycolytic kinase PGK1 and oxidative stress sensor DJ1. We performed structure–activity studies on the series to develop a photoaffinity probe to deconvolute the cellular targets. In vitro binding and structural analyses confirmed these targets, suggesting that PGK1/DJ1 interact, which we confirmed by immunoprecipitation. Glucose homeostasis and oxidative stress are linked to telomere signaling and exemplar compound CRT0063465 blocked hypoglycemic telomere shortening. Intriguingly, PGK1 and DJ1 bind to TRF2 and telomeric DNA. Compound treatment modulates these interactions and also affects Shelterin complex composition, while conferring cellular protection from cytotoxicity due to bleomycin and desferroxamine. These results demonstrate therapeutic potential of the compound series.

## Introduction

Telomere attrition, genomic instability and cellular senescence are hallmarks of aging [1]. Mammalian telomeres are nucleoprotein structures comprising repeats of the sequence TTAGGG, bound by sequence-specific and other factors which form a highly specialized chromatin environment [2]. In normal somatic cells the end replication problem, oxidative stress, and processing by nucleases cause progressive telomere shortening during each round of cell division, ultimately compromising protection at critically shortened telomeres causing DNA damage signaling and senescence [3].

The shelterin complex, comprising TRF1, TRF2, POT1, TIN2, TPP1, and RAP1, is the fundamental controller of telomere protection and its composition and activities have been extensively reviewed [4]. Shelterin proteins play diverse roles in length regulation, telomerase recruitment, and suppression of DNA damage responses and inappropriate repair. However, in aged normal cells with critically shortened telomeres these protective functions of shelterin are abrogated. Telomere dysfunction also results from polymorphism or mutation of telomerase or shelterin components. These variants associate with cancer susceptibility, idiopathic pulmonary fibrosis, and several accelerated aging syndromes, most notably dyskeratosis congenita [5], [6], [7], [8], [9], [10]. Restoring telomere protection is therefore an important goal in aging research and cancer prevention.

Besides pathways of telomere homeostasis, metabolic defects are also powerful drivers of cell aging. Mitochondrial dysfunction and anabolic signaling/ loss of nutrient sensing accelerate cell aging, while dietary restriction increases organismal longevity and decreases age-related pathologies in a variety of models [1]. These observations have led to the suggestion that development of pharmacological mimetics of caloric restriction is a promising avenue for anti-aging drug discovery [11]. Among these agents, glycolysis inhibitor 2-deoxyglucose (2-DG) attracted early attention and was shown to increase longevity in nematodes. In rats, 2-DG produced caloric restriction-like effects including decreased serum insulin and body temperature in a rat study. Although 2-DG proved relatively toxic, glycolysis remains an interesting target for development of these agents [12].

One mechanism for promotion of aging by glycolysis involves production of advanced glycosylation end products via glycation reactions with glycolytic intermediate species including methylglyoxal. In turn, glycation damage to DNA, lipids and proteins can cause mitochondrial dysfunction, accelerating oxidative stress [13]. Recently, however, several studies have also emerged linking glycolysis and mitochondrial function with telomere homeostasis pathways. Glucose restriction has been shown to decrease hTERT expression and telomerase activity [14]. Extra-nuclear hTERT has also been reported to influence glucose uptake by interacting with glucose transporters [15].

We have previously applied cell based- and virtual-screening approaches for target and compound identification to selectively modulate telomere signaling pathways [16], [17], [18], [19]. We now report characterization of CRT0063465, a novel pyrazolopyrimidine compound that emerged from cell-based screens of telomere signaling. CRT0063465 targets the nucleotide binding site of glycolytic enzyme phosphoglycerate kinase 1 (PGK1). The compound also appears to bind the multifunctional cytoprotective glyoxalase protein PARK7/DJ1. We show here that DJ1 and PGK1 form a previously unknown complex in cells.

In further investigating cellular activity of CRT0063465, we found it acts to block telomere erosion under hypoglycemic stress independently of telomerase activity. These results prompted us to investigate potential interactions of PGK1/DJ1 with telomeres. We show that both proteins bind TRF2 and telomeric DNA. These interactions are modulated by hypoglycemia and CRT0063465 treatment. Intriguingly, multiple other glycolytic enzymes also associate with TRF2. In unstressed conditions, proteomic analysis indicated that CRT0063465 causes shelterin remodeling through recruitment of TPP1, TIN2 and exonuclease Apollo to TRF2. Together, these results suggest that telomere signaling and energy metabolism may be more tightly connected than previously supposed. Beyond effects on telomere length, CRT0063465 also confers partial resistance to several DNA damaging treatments, suggesting future strategies for evaluation of its therapeutic potential.

## Materials and Methods

### Cells and Compounds

Cells were A2780 ovarian adenocarcinoma cells and HCT116 colorectal cancer cells. Before synthesis of CRT0105481, structure–activity studies indicated C4-carboxylate (CRT0063465) and ester moieties (CRT0063459) were optimal. Small substituents on the CRT0063465 C2-aryl ring para-position (CRT0063465, CRT0063466) were better tolerated than larger substituents (CRT0163463). Other ring substitution positions were poorly tolerated (CRT0098928). These studies are detailed in PubChem AID1259345. Un-labeled phenylazide derivative (CRT0066127) was tested before synthesis of tritiated version CRT0105481.

### Synthetic Routes

**Figure 1:**
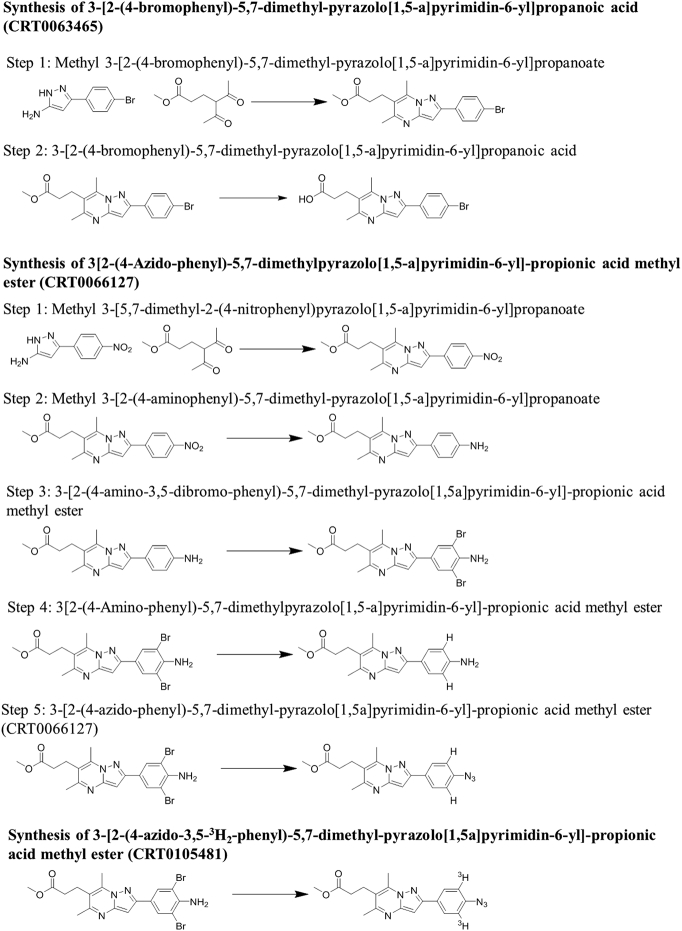
Schema for synthesis of CRT0063465, CRT0066127, and tritiated photoaffinity cross-linking derivative CRT0105481. Experimental details are given in Supplementary File 1.

Syntheses are outlined schematically in Figure 1, below. Detailed information and synthesis data for each step is provided in Supplementary File 1.

### Photoaffinity Cross-Linking and Autoradiography

Cells were incubated for 30 minutes in 10 nM or 100 nM CRT0105481 then exposed to 120 mJ 254 nm UV light using a Stratalinker (Agilent, Santa Clara, CA) and harvested for 2D gel analysis and autoradiography. Gels were stained with Simply Blue Safe Stain (Thermo Fisher Scientific, York, UK), infused with En3hance autoradiography enhancer reagent (Perkin Elmer, Warrington, UK), dried, and incubated with radiographic film for 1 week (100 nM CRT0105481 gels) or 1 month (10 nM CRT0105481 gels) at −70 °C. Labeled spots were excised for LC–MS/MS analysis. Recombinant human proteins PGK1 (ab211320) and DJ1 (ab51198) for in vitro labelling were obtained from Abcam (Cambridge, UK).

### Two-Dimensional Electrophoresis

First dimension isoelectric focusing used an IPGphor (Amersham Biosciences, UK) with surface temperature 20 °C and maximum current 50 μA/strip. Proteins (500 μg) were precipitated with cold acetone (1:4, overnight at −20 °C) and resuspended to final volume 350 μl with rehydration solution containing 8 M urea, 2% 3-[(3-cholamidopropyl)dimethylammonio]-1-propanesulfonate (CHAPS), 10 mM 1,4-dithioerythritol (DTE), 2% resolytes pH 3–10, trace bromophenol blue (BB). Samples were applied to the IPG-strip (24 cm pH 3–10 nonlinear; Amersham Biosciences, UK) by both passive (1 hr. @ 20 °C) and active rehydration (overnight, 50 V @ 20 °C). Focusing parameters were: step 1 – step hold 500 V for 1 h; step 2 – gradient 1000 V for 1 h; step 3 – gradient 8000 V for 3 h, step 4 – hold 8000 V for approx. 63 kV h. Focused proteins were reduced and alkylated during the equilibration step immediately before transfer to the second dimension. IPG strips were first covered with equilibration solution (50 mM Tris–HCl buffer (pH 8.8), 6 M urea, 30% w/v glycerol, 2% w/v SDS, and a trace of BB) containing (1% w/v) DTE for 15 min. This solution was replaced with equilibration solution containing (4% w/v) iodoacetamide for 15 min. Equilibrated strips were separated across 5–20% gradient SDS-PAGE gels (256 mm L × 200 mm W, (optigel,sigma)), using a Ettan DALTtwelve system and standard SDS buffer (Invitrogen). Gels were run at a 500 V (limits 350 A / 17 W per gel) until the dye front left the gel (approximately 8 h).

### 2D Protein Spots LC–MS Analysis

Excised protein spots were washed by sequential dehydration/hydration steps alternating between acetronitrile (MeCN) and 50 mM ammonium bicarbonate (AB). Trypsin digests were performed at 37 °C for 90 min followed by extraction (0.1% trifluoroacetic acid and 5% MeCN), and concentration. Separation was performed on Ultimate 3000 nanoLC (Dionex, Thermo Fisher Scientific, York, UK). Samples were pre-concentrated on a PepMap100 trap column 5% MeCN and 0.1% formic acid, and separated on a PepMap 100 C18 analytical column using a gradient of 80% MeCN, 0.1% formic acid; 8–25% for 70 minutes, then 25–50% for 30 minutes at 300 nl/min. Samples were injected into a Q-Star mass spectrometer (Applied Biosystems). MS data were acquired by 1.5-s survey scan (mass range 400–1200 Da) and data-dependent MS/MS of the three most intense ions with charge state +2 to +4, for 2 s. These were then excluded from acquisition for 30 s. Mass spectra recalibration used an in-house script, searched against UniProtKB-Swiss-Prot (Ver. 54.3) using Mascot 2.0 (Matrix Science) with mass tolerance of 12–30 ppm for parent and 0.12 Da for fragments ions. The protein identification list was filtered using non-MUDPit scoring.

### TRF2 Pull-Down LC–MS Analysis

Gel lanes were excised to six approximately equal sized bands and destained by successive washing (10 min, 30 °C) in water, 50% acetonitrile, 0.1 M triethylammonium bicarbonate (TEAB, pH 8.5) and 50 mM TEAB in 50% acetonitrile. Gel was dehydrated in 100% acetonitrile and dried by Speedvac (Thermo Fisher Scientific, York, UK) before rehydration in 25 mM TEAB containing 5 μg/ml trypsin overnight (30 °C). Digests were extracted sequentially with one volume of 100% acetonitrile (15 min, 30 °C) and one volume of 25% acetonitrile and 1.25% formic acid (15 min, 30 °C). Supernatants were pooled and evaporated. Dried peptides were re-dissolved in 5% acetonitrile/0.25% formic acid. Reversed-phase LC–MS/MS analysis was performed on LTQ-Orbitrap Velos coupled to a Proxeon Easy-LC (Thermo Fisher Scientific, York, UK). Peptide mixtures were loaded onto C18 guard columns (1.9 μm; 0.1 × 20 mm) and separated on a C18 in-house packed emitter (1.9 μm; 0.075 × 150 mm) over a 55 min linear gradient (5% to 45% B. A: 2% acetonitrile 0.1% formic acid B: 80% acetonitrile 0.1% formic acid). The Orbitrap survey scan analysis (m/z 350 to 1600) was at 60,000 resolution and the top 10 ions in each duty cycle were selected for MS/MS in the LTQ linear ion trap with collision-induced dissociation (CID) (normalized collision energy (NCE) 36%). Data was searched against *Homo sapiens* Swiss-Prot (148,212 entries) using Mascot (2.4.1). Mascot files were loaded into Scaffold (4.3.4). The mass spectrometry proteomics data have been deposited to the ProteomeXchange Consortium [20] via the PRIDE [21] partner repository with the dataset identifier PXD011356 and https://doi.org/10.6019/PXD011356.

### Crystallography

Crystals of PGK1 in complex with 3-phosphoglycerate and MgCl_2_ were grown as previously described [22]. A single crystal was soaked in 5 mM CRT0063465 for 3 days and flash-frozen in liquid nitrogen using LV cryo-oil (MiTeGen) as cryoprotectant. Data collected to 1.9 Å resolution at 100 K on beamline I-04 at the Diamond synchrotron (Harwell, UK) were processed using iMosflm [23] and CCP4 suite [24]. The structure was solved using PHASER [25] and PGK1/D-ATP as the search model (PDB 2ZGV). The model was rebuilt using COOT [26] and refined using REFMAC5 [27]. Table 1 presents data collection and refinement statistics. The PGK1/CRT0063465 structure is deposited in PDB (accession code 5NP8).

**Table 1:** Crystallography data collection and refinement statistics. Values in parenthesis are for the highest resolution shell

Data collection		Refinement	
Space group	*P*2_1_	Resolution (Å)	28.00-1.90
Cell parameters a, b, c (Å); β (°)	35.9, 103.9, 50.7; 99.1	No. reflections	26,787
Resolution (Å)	30.0–1.9 (2.0–1.9)	*R*_work_/*R*_free_	0.168/0.222
*R*_meas_	0.076 (0.387)	No. atoms (protein/ligand/water)	2977/23/234
<I/σI>	18.5 (5.9)	B factors (protein/ligand/water)	25/41/32
Completeness (%)	98.1 (96.7)	R.M.S. deviations	
Redundancy	6.7 (6.5)	Bond length (Å) Bond angle (°)	0.019 1.97

### In Silico Structural Modeling

Monomeric DJ1/PGK1 complex structure was derived by overlaying the CRT0063465-PGK1 costructure with the C106-sulfinic acid-bound DJ1 structure, and with PDB DJ1 structure (1P5F.PDB), aligning protein units through overlay of bound CRT0063465 (CRT0063465 fragment in the case of the DJ1 structure). The resulting complex, containing only bound CRT0063465 from the PGK1 crystal structure, showed protein–protein steric clashes, so the DJ1 structure was subjected to local energy minimization in loop regions contacting PGK1. The full complex underwent constrained energy minimization, keeping the ligand structure rigid. To construct the DJ1 dimeric complex, two units of the minimized monomeric complex were overlain with the dimer DJ1 crystal structure 1SOA.PDB, using DJ1 sequence. Structural alignment guided positioning of protein units. Constrained minimization was conducted on the final dimeric complex, again keeping both ligand structures fixed. Calculations were performed using Molecular Operating Environment (MOE) 2010.10 from Chemical Computing Group Inc., using the Amber99 forcefield.

### Surface Plasmon Resonance

SPR was performed using a ProteOn™ XPR36 system (BioRad, Hemel Hempsted, UK). > 4000 RU of his-tagged PGK1 at concentrations of 80 μg/ml and 25 μg/ml was immobilized by amine coupling onto a GLH sensor chip. Subsequently, CRT0063465 was injected over the chip in a range of concentrations to determine K_d_ values. Purified recombinant PGK1 was obtained from Crelux (Martinsried, Germany).

### Immunoprecipitation

Immunoprecipitations were performed using Dynabeads (Thermo Fisher Scientific, York, UK). Antibodies (10 μg) were coupled to M270 beads according to the manufacturer's protocol. Beads were washed in C1 buffer then C1-diluted antibodies added. Buffer C2 was added and reactions incubated for 18 h at 37 °C. HB and SB washes contained 0.1% Tween-20 (Sigma-Aldrich, Dorset, UK). Before use, beads were washed in PBS containing 0.1% BSA. Lysates were prepared in kit extraction buffer supplemented with EDTA-free protease inhibitor cocktail tablets (Roche Diagnostics Ltd., West Sussex, UK) and 100 mM NaCl. Immunoprecipitations were performed for 30 minutes at 4 °C, then beads were washed and eluted. Antibodies were ab90787, PGK1; ab18257, DJ1; and ab13579, TRF2 (Abcam, Cambridge, UK). Experiments were performed twice.

### Western Blotting

Cytoplasmic and nuclear extracts were prepared with NE-PER extraction reagents (Thermo Fisher Scientific, York, UK). Whole cell extracts were prepared with Dynabeads lysis buffer (Thermo Fisher Scientific, York, UK). Proteins were separated by SDS-PAGE, blotted onto PVDF filter (Millipore, Watford, UK) and blocked overnight in PBS-T containing 5% non-fat dried milk. Antibodies were ab67335, PGK1; ab131591, DJ1; ab4182, TRF2; and ab97433, Ku80 (Abcam, Cambridge, UK). HRP-conjugated secondary was detected using ECL HRP detection reagents (Amersham Pharmacia, Buckinghamshire, UK). Experiments were performed at least twice.

### Telomerase Activity Assays

TRAPeze XL kits were used for TRAP assay (Millipore, Watford, UK). Cells were lysed in CHAPS buffer and protein concentrations estimated by Bio-Rad assay (BioRad Laboratories Ltd., Hemel Hempstead, UK). Protein (0.5 μg) was mixed with TRAPeze reaction mix containing TS primer, fluorescein labeled RP primer, control template and sulforhodamine labeled control K2 primer. Controls included no-telomerase, no-Taq, and heat-treatment. 30 °C extension products were detected by Q-PCR in triplicate using Chromo4 equipment (BioRad Laboratories Ltd., Hemel Hempstead, UK). Total product was measured against TR8 standards. Experiments were performed three times in triplicate.

### Telomere Restriction Fragment Analysis

Telomere length assays were performed using the teloTAGGG kit (Roche Diagnostics Ltd., West Sussex, UK). One microgram of genomic DNA was digested with HinfI/RsaI. Digests were separated by gel electrophoresis and blotted onto positively charged membrane (Roche Diagnostics Ltd., West Sussex, UK). Membranes were UV cross-linked, baked at 120 °C and washed in 2×SSC solution. Hybridization of DIG-labeled telomeric probe was performed using buffers and probe provided. Membranes were washed, probed with alkaline phosphatase-conjugated anti-DIG and exposed to CDP-star. Experiments were performed twice.

### Chromatin Immunoprecipitation

Cells cultured for 1 week in the presence of physiological glucose or hypoglycemia and 10 nM CRT0063465 were harvested at 70%–80% confluence, fixed in formaldehyde and lysed in SDS buffer with protease inhibitors. Chromatin fragments (500 bp-1 kb) were generated by sonication using a Branson S25OD sonifier (Branson Ultrasonics Corp., Danbury, CT). Antibodies were Ab13579, TRF2; Ab67355, PGK1; Ab131591, DJ1 (Abcam, Cambridge, UK). Each assay included no-antibody control. Telomeric DNA was detected by Q-PCR in triplicate using sybr green and Chromo4 equipment (BioRad Laboratories Ltd., Hemel Hempstead, UK). Primers were 5′-CGGTTTGTTTGGGTTTGGGTTTGGGTTTGGGTTTGGGTT-3′ and 5′-GGCTTGCCTTACCCTTACCCTTACCCTTACCCTTACCCT-3′. Experiments were performed three times with qPCR in triplicate on each occasion.

### Electromobility Shift Assays

5-mer telomere oligonucleotide 5′-(TTAGGG)_5_-3′ (Sigma-Aldrich, Dorset, UK) was 3′-biotinylated using Pierce reagents (Thermo Fisher Scientific, York, UK) and hybridized to complementary sequence oligonucleotide. Nuclear extracts were prepared using NE-PER reagents (Thermo Fisher Scientific, York, UK). EMSAs were performed using LightShift Chemiluminescent EMSA kit (Thermo Fisher Scientific, York, UK). Reactions included 2.5% glycerol, 5 mM MgCl_2_, 50 ng/μL poly(dI^**.**^dC), 0.05% NP40 and 20 fmol labeled probe with or without 200-fold excess of unlabeled competitor or 2.5 μg nuclear extracts. Complexes were electrophoresed at 4 °C on 6% DNA retardation gels (Thermo Fisher Scientific, York, UK) and blotted onto nylon membrane (Roche Diagnostics Ltd., West Sussex, UK). Label detection used Chemiluminescent Nucleic Acid Detection Module kit reagents (Thermo Fisher Scientific, York, UK.

### Real-Time Kinetic Growth Assays

Prior to cell seeding in duplicate wells of xCELLigence 96 well E-plates (ACEA Biosciences, CA), initial medium only reads were performed in an xCELLigence RTCA Single Plate instrument (ACEA Biosciences, CA). Plates were removed from the instrument and cells were seeded at 10,000 per well and allowed to attach for 30 minutes prior to continuing with the read programme. Twenty-four hours post-seeding, the run was paused and medium was replaced with treatment medium. Growth was then monitored every 6 h for 24 h post-treatment and every 24 h for a further 72 h. Experiments were performed twice in duplicate.

### Statistical Analysis

Statistical analyses were performed in Matlab (Mathworks, Cambridge, UK).

## Results

### A Novel Pyrazolopyrimidine Ligand of PGK1 and DJ1

**Figure 2:**
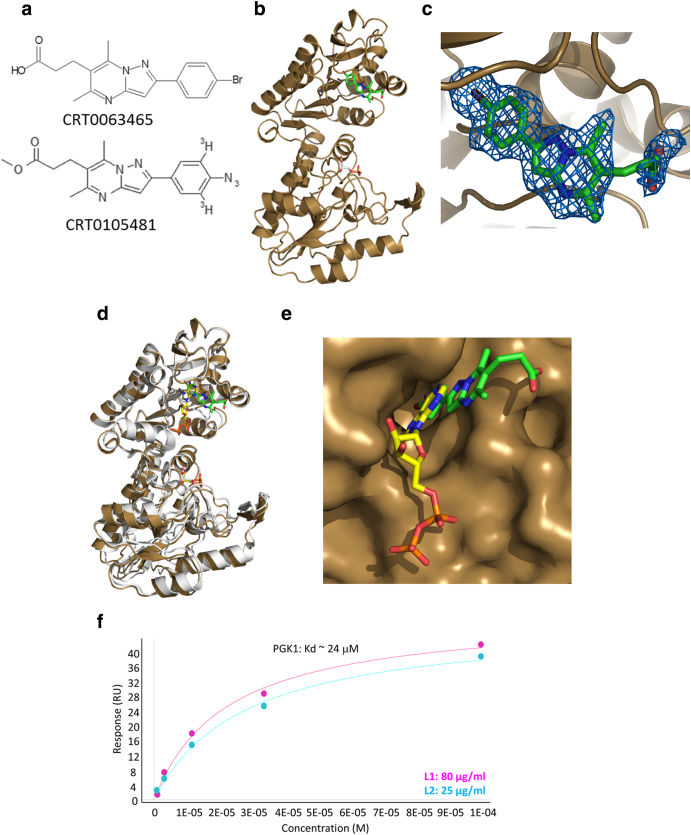
CRT0063465 binds to phosphoglycerate kinase 1 promoting a conformational shift. (a), molecular structures of CRT0063465 and the radiolabeled photoaffinity crosslinking analogue CRT0105481. (b), Binding modes of CRT0063465 and 3-phosphoglycerate to PGK1. Crystal soak-in experiments were performed with CRT0063465 at 5 mM concentration and electron density data were collected at 1.9 angstroms. (c), Close-up of CRT0063465 binding mode to PGK1 nucleotide binding site, overlaid with compound electron density. (d), CRT0063465 promotes a conformational shift in PGK1. The overlaid ribbon models show conformations of PGK1 bound to3-phosphoglycerate (yellow, lower lobe) and to either CRT0063465 (green compound, brown ribbon) or to ADP in the “closed” form (yellow compound bound to upper lobe, white ribbon). (e), Close-up of CRT0063465 binding mode to PGK1 nucleotide binding site, overlaid with ADP structure. (f) Surface Plasmon Resonance dose–response curves for CRT0063465 binding to PGK1. PGK1 was immobilized on a GLH sensor chip at either 80 μg/ml or 25 μg/ml and CRT0063465 was injected at a range of concentrations. Curve fitting was performed using ProteOn Manager software to determine K_d_ value.

We previously reported development of complementary cell-based assays focused on regulation of the telomerase gene promoters [17], [19] and cell survival after enforced telomere uncapping [2]. These were used to probe 1000 and 4500 diverse compounds, respectively. Full assay methodology and screening results are reported in PubChem bioassays AID1259240 and AID1259312. Confirmatory assays and structure activity investigations are also presented in additional bioassays referred to throughout the text. Characterization of hits in our initial screens in A2780 ovarian cancer cells identified a pyrazolopyrimidine compound with activity in both assays (AID1259345 and AID1259347), suggesting a favorable profile as a potential regulator of immortalisation pathways (CRT0063465, Figure 2*a*). To facilitate target identification, structure–activity relationships (SAR) around the pryrazolopyrimidine scaffold were probed, leading to development of a tritiated phenylazide derivative for use as an in-cell photoaffinity cross-linking probe (CRT0105481, Figure 2*a*). Several of the derivatives developed during SAR studies are detailed under PubChem bioassay AID1259345 and summarized in the methods section. Synthesis data for exemplar compounds are also given in Supplementary File 1.

To identify targets of the series, A2780 cells were treated with CRT0105481 at 10 nM or 100 nM for 30 minutes. UV-crosslinking was performed and proteins harvested for 2D-GE and autoradiography. Radiolabeled spots excised from the 2D gels were analyzed by LC–MS to determine their composition. These experiments revealed that CRT0105481 bound to 2 cellular proteins: glycolytic kinase PGK1 and multifunctional stress response protein PARK7/DJ1 (Figure S1 in Supplementary File 2). Commercially available purified recombinant preparations of these were obtained to confirm binding. Incubation with CRT0105481 at 10 nM or 100 nM followed by UV crosslinking, SDS-PAGE and autoradiography demonstrated dose-dependent binding to both purified targets (Figure S2 in Supplementary File 2).

To investigate binding modes of the unlabeled parent compound CRT0063465 to these proteins, X-ray crystallography was performed. A PGK1- CRT0063465 co-structure was obtained at 1.9 Å resolution (Figure 2*b*). CRT0063465 binds an “open” conformation of PGK1 equivalent to the ternary complex with 3-phosphoglycerate and ADP (PDB 2XE7). Data collection and refinement statistics are presented in Table 1. Binding is perpendicular to the nucleotide binding site (Figure 2, *b* and *c*). The compound bromo-phenyl moiety occupies the same position as the nucleotide adensosine ring. The aryl ring is co-planar with the pyrrolopyrimidine scaffold and the para-substituted bromine engages a deep hydrophobic pocket. The carboxylate moiety on the distal end of the pryrazolopyrimidine scaffold is solvent-exposed and does not interact via any polar or hydrogen bonding interactions with PGK1 (Figure 2, *d* and *e*). CRT0063465 makes no hydrogen-bonding hinge region interactions. This mode would be unusual for an ATP-competitive kinase inhibitor, which normally bind mainly through this mechanism [28].

To provide further evidence of binding to PGK1, SPR measurements were performed against immobilized purified PGK1 at a range of CRT0063465 concentrations. Measured K_d_ was approximately 24 μM (Figure 2*f*). It is possible that this relatively high value results from conformational changes associated with PGK1 immobilization, since CRT0105481 bound PGK1 in vitro and in cells at nM concentrations. Moreover, in a metabolomics screen following treatment of HCT116 colorectal cancer cells, which are known to exhibit a highly glycolytic phenotype [29], with 10 nM CRT0063465 for 2 h, we observed significant upregulation of a 170 Da species, identified with high confidence as immediate upstream glycolysis metabolite(s) glyceraldehyde 3-phosphate and/or its isomer dihydroxyacetone phosphate (Figure S3 and S4, and table S1 in Supplementary File 2). These results indicate that the compound does indeed block glycolytic flux in this cell line at nanomolar concentrations. Several other species were also significantly affected including uracil, 16-hydroxypalmitate, and various fatty acid intermediates.

**Figure 3:**
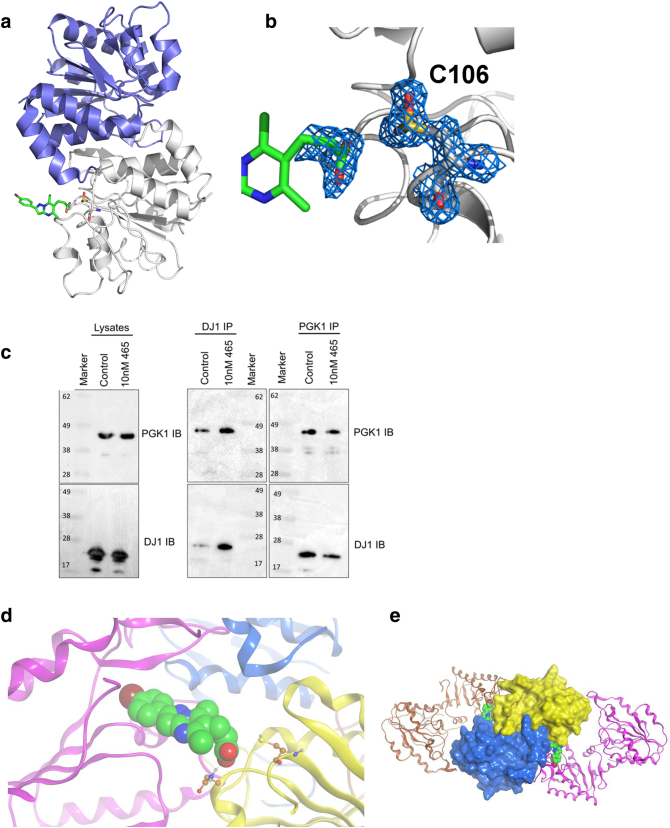
Identification of a complex between PGK1 and DJ1 modulated by CRT0063465. (a), Putative mode of binding between CRT0063465 and DJ1. (b) Close up of proposed binding mode, showing electron density of the oxidized C106 residue and adjacent residual electron density overlaid with the CRT0063465 carboxylate moiety. (c), Detection of a cellular PGK1/DJ1 complex. A2780 cells were treated with CRT0063465 or vehicle for 1 h and harvested for immunoprecipitation of either target. Immunoblots were performed to detect the presence of each protein in unprecipitated cell lysates or in immunoprecipitates. The experiment was performed twice. Representative blots are shown. (d), Close-up of candidate interfacial binding mode. Interaction between CRT0063465 and oxidized C106 in DJ1 leaves the PGK1 hydrophobic tunnel accessible to the bromoaryl portion of CRT0063465. (e), Molecular docking simulation of proposed 2:2:2 complex between PGK1, DJ1 and CRT0063465. The compound-bound PGK1 and DJ1 X-ray structures were aligned along the axis of CRT0063465 by overlaying the compound scaffold in either structure.

Co-crystallization of DJ1 with the compound was unsuccessful. However, soaking experiments using DJ1 apoform crystals grown as previously described [30], revealed residual electron density associated with DJ1 C106. This residue was found to be in oxidized sulfinic acid form. In cells, DJ1 C106 oxidation plays a key role in its cytoprotective function, regulating cellular localization and p53-dependent transcription [31], [32]. It is possible that the residual electron density corresponds to the compound carboxylate moiety (Figure 3, *a* and *b*). In this putative structure, the core heterocycle and aryl ring are disordered and located in the solvent-exposed region. These could not be refined; therefore, this binding mode cannot be confirmed.

However, previous studies indicate a role for this residue in diverse small molecule interactions. Landon and colleagues used the multiple solvent crystal structures method and computational fragment mapping to implicate C106 as a ligand-binding hot-spot [33]. The structure of DJ1 covalently bound to glyoxylate via C106 was also recently determined [34]. These results support our hypothesis that this residue represents the binding site for the CRT0063465 carboxylate. This proposed binding mode, involving an electrostatic interaction, is also consistent with structure–activity data in our cell based assays indicating that modification of the carboxylate to an uncharged group causes loss of activity, despite the lack of interaction of this moiety with PGK1 (Pubchem AID1259345, Figure S5 in Supplementary File 2, and Figure 2). Taken together our data indicate that the compound can bind both proteins, although the mechanism is clearest for PGK1.

### A PGK1/DJ1 Complex Modulated by CRT0063465

Recently, novel cellular protection roles for DJ1 as a glyoxalase and deglycating enzyme for both proteins and nucleic acids have been reported [35], [36], [37]. Methylglyoxal, one of the principal cellular glycating species is produced from glycolytic intermediates dihydroxyacetone phosphate and glyceraldehyde-3-phosphate, which are generated by the aldolase glycolysis reaction upstream of PGK1 [35] and were increased by CRT0063465 (Figure S3 and S4, and table S1 in Supplementary File 2). Physical interaction between DJ1 and PGK1 could localize DJ1 close to sites of methylglyoxal production. Interestingly, a previous proteomic study has also shown association of DJ1 with several other enzymes of the glycolysis pathway (though not PGK1) [38]. We therefore investigated the possibility that CRT0063465 may modulate a PGK1/DJ1 interaction.

We performed co-immunoprecipitations of both proteins in A2780 ovarian cancer cells, in which the targets were initially identified (Figure 3*c*). Cells were treated with vehicle or 10 nM compound for 1 hour prior to harvesting for immunoprecipitation. Western blotting of lysates used for immunoprecipitation revealed no significant change in levels of either protein (left panel). Reciprocal co-immunoprecipitation confirmed that PGK1 and DJ1 do indeed interact (middle and right panels). Importantly, these results confirm the existence of a previously unknown PGK1/DJ1 complex. Interestingly, CRT0063465 also led to increased pulldown of DJ1 as judged by blotting DJ1 immunoprecipitates with a different DJ1 antibody (middle panel). Correspondingly, co-immunoprecipitated PGK1 also increased. However, there was little change in levels of either protein in the PGK1 pulldowns (right panel). The effect on DJ1 immunoprecipitation might result from conformation changes associated with C106 oxidation, perhaps presenting additional epitopes for the polyclonal pulldown antibody.

To investigate the possible interaction mode of PGK1/DJ1, we performed in silico docking of both proteins using the CRT0063465-PGK1 costructure and putative C106-sulfinic acid-bound DJ1 structure, aligning the proteins along the compound axis by overlaying the CRT0063465 scaffold from each structure (Figure 3, *d* and *e*). In this model, the DJ1 homodimer (PDB 1SOA) forms the core, with C106 from each subunit positioned on opposite faces. CRT0063465 carboxylate interacts with the C106-sulfinic acid in DJ1 while the bromoaryl ring interacts with the PGK1 nucleotide binding domain hydrophobic tunnel (Figure 3*d*). CRT0063465-bound PGK1 domains are locked in the “open” conformation, each interacting in a clamp-like fashion with a single DJ1 monomer on opposite sides of the complex, giving 2:2:2 CRT0063465:DJ1:PGK1 stoichiometry (Figure 3*e*). Although this is a computational model, it is in line with the structure activity relationships observed in our screening assays (Pubchem Bioassay AID1259345 and Figure S5 in Supplementary File 2). The model is available at Model Archive (part of Protein Model Portal [39]) with accession number ma-4hhdq.

### CRT0063465 Protects Telomeres from Erosion Under Hypoglycemia

**Figure 4:**
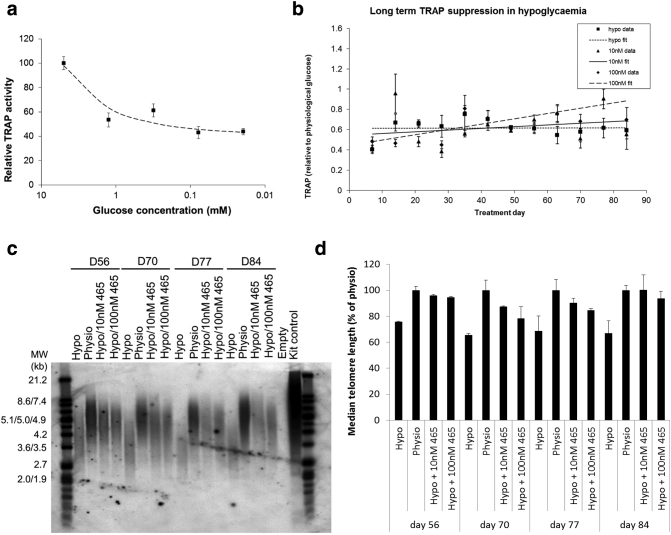
Blockade of hypoglycemic telomere erosion by CRT0063465. (a), Hypoglycemia suppresses telomerase activity. HCT116 cells were cultured for 1 week in medium containing different glucose concentrations prior to harvesting for TRAP analysis. Mean ± SEM of 3 experiments performed in triplicate. Least squares curve fitting to the exponential model was performed by using Microsoft Excel Solver. (b), CRT0063465 does not affect long term telomerase activity suppression by hypoglycemia. HCT116 cells were cultured for 84 days in medium containing 5 mM (physiological) or 500 μM (hypoglycemic) glucose in the presence of absence of 10 nM or 100 nM CRT0063465. Samples were taken weekly for TRAP analysis. Mean ± SEM of 3 experiments performed in triplicate. Least squares curve fitting to linear models was performed using Microsoft Excel Solver. (c), Telomere erosion under hypoglycemia rescued by CRT0063465. Late time point samples from the long-term culturing experiment in (b) were analyzed for telomere length by Telomere Restriction Fragment (TRF)-Southern blotting. Experiments were performed twice. A representative blot is shown. (d), Densitometry quantification of median telomere length. TRF peak signals from both repeats of the long term culturing experiment were analyzed against the lane marker peaks in GeneTools (Syngene, Cambridge, UK). Mean ± SEM of 2 experiments.

Recently, several studies have elucidated links between energy metabolism and telomere signaling. Glucose restriction has been found to decrease hTERT expression and telomerase activity [14], while extra-nuclear hTERT has also been reported to influence glucose uptake by interacting with glucose transporters [15]. CRT0063465 itself is a novel PGK1 nucleotide binding site ligand that emerged from our screens of telomere signaling. We therefore investigated whether interactions between telomeres and glycolysis could be influenced by the compound. For these experiments, we adopted glucose restriction to further manipulate glycolysis, using HCT116 colorectal cancer cells, which are known to exhibit a highly glycolytic phenotype [29]. We have also found CRT0063465 to be active in our cell-based reporter screening assay in these cells (PubChem AID1259346). We first performed telomerase activity (TRAP) assays on cells exposed to hypoglycemia. Our results agree with those of Wardi and colleagues [14], confirming that glucose restriction causes dose-dependent reduction in telomerase activity (Figure 4*a*).

To determine whether addition of CRT0063465 modifies these effects in the glucose-restricted setting, we next performed long-term culturing experiments in the presence of hypoglycemia alone, or in combination with 10 nM or 100 nM CRT0063465. Cells were fed twice-weekly with low-glucose (500 μM) medium with CRT0036465 maintained on cells throughout. Hypoglycemia alone led to a relatively stable reduction of telomerase activity over 84 days in culture (Figure 4*b*). Cells could be cultured easily under long-term hypoglycemia, though growth was substantially reduced compared to controls supplemented with physiological glucose (Figure S6 in Supplementary File 2). Treatment with CRT0063465 had no additional long-term effect either on cell growth or on total telomerase activity, as judged by the TRAP assay (Figure 4*b*).

When telomere restriction fragment Southern blots of the time-course were examined, consistent with the reduction in telomerase activity, telomere lengths were decreased in hypoglycemia relative to physiological conditions. Strikingly, however, despite the lack of effects observed on total cellular telomerase activity by CRT0063465, hypoglycemic erosion of telomeres was blocked in the presence of the compound at both 100 nM and 10 nM (Figure 4*c*). This experiment was performed twice with similar results. Quantification of both experiments indicated that median telomere length in hypoglycemic cells reduced by 35% by day 70, relative to physiological glucose conditions and similar reduction was observed at days 77 and 84. In 10 nM CRT0063465 treatments, the maximum reduction observed was only 13% on day 70, and on day 84 lengths were similar to those in the physiological condition (Figure 4*d*). Interestingly, one experiment was continued until day 147. Analysis of later time points in this experiment suggested that the effect may be durable over longer periods (Figure S7). Hence, CRT0063465 appears to block metabolic stress-induced telomere shortening in the absence of effects on telomerase.

### PGK1 and DJ1 Bind TRF2 and are Recruited to Telomeres

**Figure 5:**
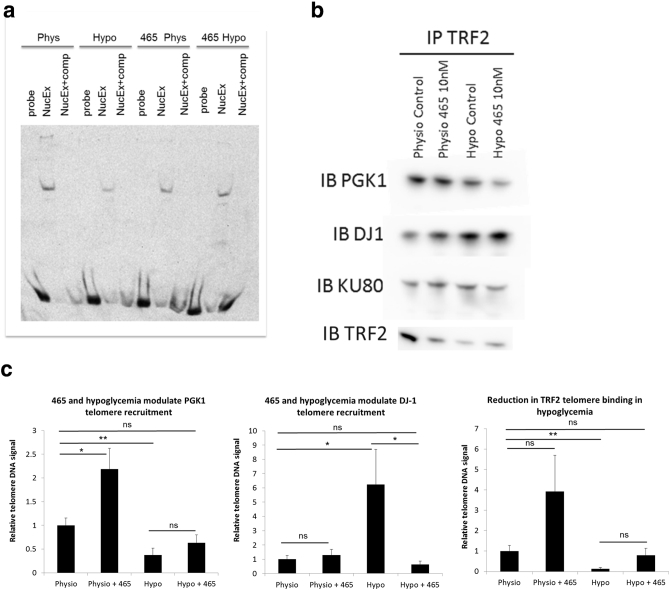
Hypoglycemia and CRT0063465 regulate interaction of PGK1/DJ1 with TRF2 and telomeres. (a), CRT0063465 rescues a nuclear telomere-binding activity in hypoglycemia. HCT116 cells were cultured for 1 week in 5 mM (physiological) or 500 μM (hypoglycemic) glucose with or without 10 nM CRT0063465. Post-treatment, nuclear extracts were prepared for gel shift analysis. Mobility of labeled probe was visualized in the absence of nuclear extract (“probe” lanes), or in the presence of 5 μg nuclear extract from each treatment condition with (“NucEx + comp” lanes) or without (“NucEx” lanes) presence of excess cold-competitor. The experiment was performed twice. A representative image is shown. (b), PGK1 and DJ1 binding to TRF2 is modulated by hypoglycemia and CRT0063465. HCT116 cells were cultured for 1 week in 5 mM (physiological) or 500 μM (hypoglycemic) glucose with or without 10 nM CRT0063465. Co-immunoprecipitation of TRF2 was performed using Dynabeads. Immunoprecipitates were blotted for the presence of DJ1, PGK1, Ku80 and TRF2. The experiment was performed twice. A representative blot is shown. (c), PGK1 and DJ1 differentially bind to telomeric DNA under hypoglycemia and CRT0063465 treatment. HCT116 cells were cultured for 1 week in physiological or hypoglycemic conditions with or without 10 nM CRT0063465 prior to chromatin immunoprecipitation using the antibodies shown. Mean ± SEM of 3 experiments with qPCR performed in triplicate.

Since CRT0063465 blocked hypoglycemic telomere erosion without affecting telomerase activity, we investigated whether the mechanism involves direct modulation of telomere signaling. We first examined effects of hypoglycemia and compound treatments on telomere binding complexes in vitro. HCT116 cells were treated for 1 week in the presence or absence of hypoglycemia and 10 nM compound. After treatment, nuclear extracts were harvested and incubated with DIG-labeled telomere sequence probe for gel-shift analysis. Figure 5*a* shows formation of a specific complex which was competed in the presence of excess cold telomere probe. In nuclear extracts from cells grown under physiological glucose, CRT0063465 had no observable effect on this complex. However, reduced levels were observed in hypoglycemic nuclear extracts. These results suggest that hypoglycemia affects a nuclear telomere sequence-directed DNA-binding activity in vitro. Interestingly, treatment with CRT0063465 under hypoglycemia restored levels of the complex. Thus, the telomere length regulation effects observed with hypoglycemia and CRT0063465 treatment may involve regulation of telomere binding factors.

We investigated this hypothesis by co-immunoprecipitation and western blotting of the core shelterin factor TRF2 in HCT116 cells treated in the presence or absence of hypoglycemia and 10 nM CRT0063465. Levels of TRF2 were reduced in its own immunoprecipitates by all treatments (Figure 5*b* left panels, bottom). Immunoblotting of the TRF2 immunoprecipitates with PGK1 and DJ1 confirmed that both proteins also interact with TRF2. PGK1 levels were also reduced in hypoglycemic conditions. However, DJ1 levels were strongly increased, suggesting possible recruitment to the shelterin complex under hypoglycemia.

We next investigated whether PGK1 and DJ1 proteins are localized to telomeres by chromatin immunoprecipitation and telomere QPCR in cells treated in the presence or absence of hypoglycemia and CRT0063465. Telomeric DNA was detected in the immunoprecipitates of both proteins under physiological conditions and without compound treatment, indicating that they are present at telomeres in the HCT116 cancer cell line (Figure 5*c*). Although glycolysis is generally considered to be cytosolic, nuclear localization of glycolytic proteins including PGK1 has previously been reported [40], [41]. Western blotting confirmed both proteins are present in cytoplasm and nucleus under all treatment conditions in these cells (Figure S8 in Supplementary File 2).

Moreover, binding profiles of both proteins at telomeric DNA were modulated by treatment (Figure 5*c*). For PGK1, 10 nM CRT0063465 increased telomere association under physiological glucose conditions. Interestingly, while hypoglycemia reduced this association, levels of hypoglycemic telomere PGK1 binding were not significantly different from controls on treatment with CRT0063465. Examination of TRF2 telomere association under these treatments showed a similar profile to PGK1. Levels of telomere-bound TRF2 were substantially reduced under hypoglycemia, but were not significantly different from physiological controls when hypoglycemic cells were treated with CRT0063465. For DJ1, compound treatment did not significantly affect telomere association under physiological conditions. However, hypoglycemia led to strong recruitment, ablated by the addition of compound. Hypoglycemic telomere recruitment may reflect increased binding to TRF2, as detected in the co-immunoprecipitation experiments (Figure 5*b*). However, unlike DJ1-telomere association, the DJ1-TRF2 interaction above was not ablated by CRT0063465.

### CRT0063465 Modulates the Composition of Shelterin

Together, these results demonstrate that both PGK1 and DJ1 interact with a core component of shelterin and localize to telomeres. Further, these associations can be modulated by hypoglycemia and by CRT0063465. However, severe hypoglycemia is an artificial condition. To determine whether the compound has other effects on TRF2 complexes under unstressed conditions, we treated HCT116 cells grown in physiological glucose with vehicle or 10 nM CRT0063465 and immunoprecipitated TRF2. LC/MS was then performed to identify other interacting partners in each condition.

**Figure 6:**
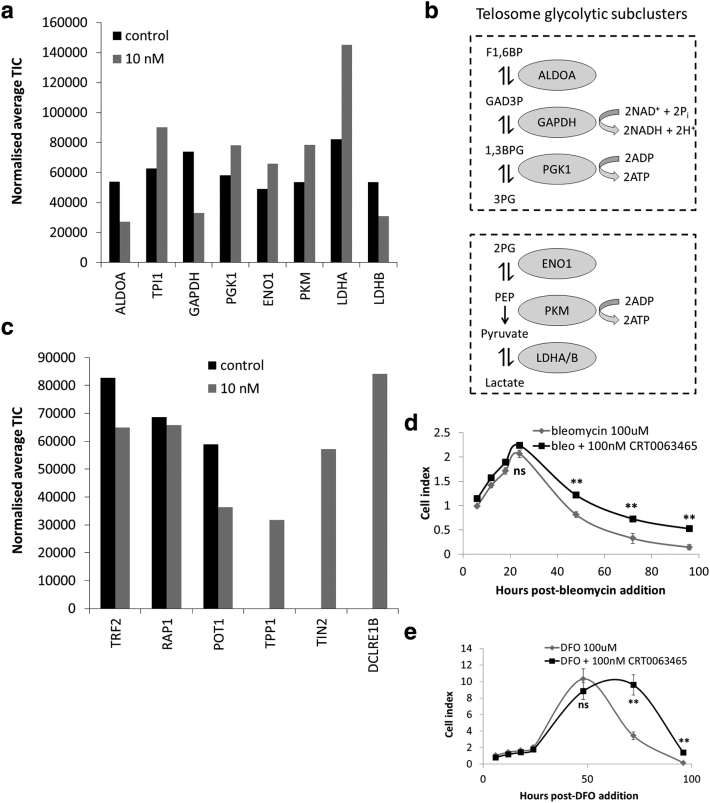
Shelterin modulation and cytoprotection by CRT0063465 in physiological conditions. (a) Identification of multiple glycolysis pathway proteins in TRF2 complexes under physiological glucose conditions. TRF2 immunoprecipitates obtained after 1 week treatment of HCT116 cells in 5 mM glucose with or without 10 nM CRT0063465 were analyzed by LC/MS and average total ion counts (TIC) were obtained for each protein in each condition using Scaffold 4. (b) Schematic representation of TRF2-associated subclusters of the glycolysis cascade identified in physiological conditions. (c) Identification of shelterin components identified in TRF2 immunoprecipitates under physiological glucose culture conditions. TRF2 immunoprecipitates were obtained after 1 week treatment in 5 mM glucose with or without 10 nM CRT0063465 were analyzed by LC/MS and average total ion counts (TIC) were obtained for each protein in each condition using Scaffold 4. (d), Protection from bleomycin-induced cytotoxicity. A2780 cells were seeded into duplicate wells of xCELLigence 96 well plates and allowed to attach prior to addition of treatments shown. Plate reads were performed every 6 h for 24 h then every 24 h for a further 72 h. Mean ± SEM of 2 experiments performed in duplicate. (e), Delayed DFO-induced cytotoxicity. A2780 cells were seeded into duplicate wells of xCELLigence 96 well plates and allowed to attach prior to addition of treatments shown. Plate reads were performed every 6 h for 24 h then every 24 h for a further 72 h. Mean ± SEM of 2 experiments performed in duplicate.

Interestingly, in addition to PGK1, multiple other glycolysis proteins were also detected in the TRF2 complexes. ALDOA, TPI, GAPDH, ENO1, PKM and LDHA/B were all present in physiological conditions (Figure 6, *a* and *b*), though upstream components HK, PGI and PFK were not. In effect, the detected proteins represent two distinct and potentially functional sub-clusters of the glycolysis cascade, disconnected by the absence of PGAM1 (Figure 6*b*). Notably, each sub-cluster is potentially ATP-generating. Quantification of areas under the peaks of each spectrum suggested most of these were increased in TRF2 complexes by compound treatment. However, changes were relatively small in most cases.

We also examined the shelterin components present in the TRF2 immunoprecipitates (Figure 6*c*). In untreated cells, TRF2 itself, RAP1, and POT1 were detected although TRF1, TPP1 and TIN2 signals were absent from the untreated condition. In the treated condition, TRF2 levels were slightly decreased as previously observed in Figure 4. Interestingly, however, signals for TPP1, TIN2 and, additionally, the 5′-exonuclease Apollo (DCLRE1B) were present following treatment with CRT0063465. Thus, CRT0063465 treatment appears to modulate the composition of shelterin complexes in unstressed cells.

Given these effects of the compound, it is of interest to examine potential protective effects beyond the setting of low glucose, using alternative treatments that may model telomeropathies. For example, it was recently shown that small molecule telomerase activator compound GRN510 suppresses bleomycin-induced lung injury in a mouse model of idiopathic pulmonary fibrosis [42]. To determine whether CRT0063465 has similar protective effects against bleomycin-induced cytotoxicity, we performed kinetic assays using the xCELLigence platform [43] to monitor real-time growth of A2780 cells treated with 100 μM bleomycin and 100 nM CRT0063465 (Figure 6*d*). We began monitoring growth at 6 h post-treatment. At 24 h post-treatment the cell index of both bleomycin/vehicle- and bleomycin/CRT0063465-treated cells increased by approximately 2-fold. At subsequent time-points up to 96 h, cell indices of both treatments decreased. However, a clear protective effect of CRT0063465 was observed at all later times. At 96 h, the cell index of bleomycin/vehicle-treated cells decreased to only 0.14, while the index of bleomycin/CRT0063465-treated cells remained 3.7-fold higher (0.52). Thus, the activity of CRT0063465 in modulating telomere composition independently of telomerase may have similar effects as telomerase reactivation in protection against bleomycin-induced toxicity.

Finally, we investigated potential for the compound to protect against cytotoxic stress due to iron chelation. Desferoxamine (DFO) is an iron-chelator widely used as a hypoxia mimetic due to its ability to stabilize HIF1α (leading, among other effects, to increased glucose uptake and glycolysis) [44]. However, high concentrations/prolonged treatments result in depletion of cellular iron pools required for ribonucleotide reductase activity, leading to inhibition of DNA synthesis and activation of DNA damage signaling [45], [46]. We performed xCELLigence experiments over 96 h on A2780 cells treated with 100 μM DFO and 100 nM CRT0063465 (Figure 6*e*). Over 48 h in culture vehicle- or CRT0063465-treated cell indices increased to 10.3 and 8.9, respectively. At 72 h, the DFO/vehicle-treated cell index decreased to 3.4, while that of DFO/CRT0063465-treated cells continued to increase to 9.6. By 96 h, both indices dropped substantially. However, the index of CRT0063465-treated cells remained around 10-fold higher than vehicle-treated cells (1.39 and 0.14, respectively). Therefore, the compound also substantially delayed cytotoxicity of high dose DFO treatment.

## Discussion

In this study we report the results of complementary cell-based screens, hit characterization, synthetic routes, and structure–activity studies leading to a novel pyrazolopyrimidine compound CRT0063465 and its analogue CRT0105481 that was used to deconvolute the cellular targets PGK1 and DJ1. We interrogated the binding mode for both proteins by X-ray crystallography, leading to the identification of a novel cellular complex between these proteins and a proposed model for the interaction. We also demonstrate for the first time that both proteins can bind to TRF2 and associate with telomeric DNA in vitro and in cells.

The lead compound CRT0063465 is a nucleotide binding site ligand of PGK1, which increased the concentration of metabolite(s) identified with high confidence as GAD3P/DHAP in a metabolomics screen, confirming activity against glycolysis. The compound modulates telomere length regulation during metabolic stress, an effect which appears to involve telomere association of PGK1/DJ1 and the critical shelterin factor TRF2. In the absence of metabolic stress, the compound caused shelterin remodeling, causing recruitment TIN2/TPP1 and the end-processing factor Apollo to TRF2 complexes. The compound also conferred protection against both bleomycin- and DFO-induced cytotoxicity, which may point to potential therapeutic uses. Bleomycin-induced lung damage has previously been used to model idiopathic pulmonary fibrosis [42]. DFO is effective in treatment of iron-overload conditions including thalassemia and sickle-cell disease, although certain tissues such as retina are sensitive to its toxic effects [47]. CRT0063465 is active in cells at low nanomolar concentration and has favorable pharmacokinetics in healthy mice (not shown).

The role of the PGK1/DJ1 complex identified here remains to be determined, though previous studies have suggested functional coupling between DJ1 and glycolysis. Interaction with PGK1 would localize DJ1 close to sites of methylglyoxal production, where its glyoxalase and deglycating activities would be most effective [35], [36], [37]. At the same time, it is plausible that this activity acts as a sensor of upstream glycolytic intermediate concentrations facilitating feedback regulation of glycolytic rate. In line with this possibility, DJ1 interacts with Foxo3a in mouse embryonic fibroblasts, resulting in stimulation of Pink1 transcription and downregulation of HIF1α and glycolytic flux [48]. In our computational model of the interaction, it is also sterically unlikely that enzymatic coupling between 1,3-bisphosphoglycerate and ADP could take place. Hence, DJ1-bound PGK1 may be catalytically inactive, which may deactivate a key step in the pathway. Our model is a starting point to guide future mutagenesis experiments. It will be interesting to determine whether DJ1 mutants that are defective in PGK1 interaction retain cytoprotective activity.

However, DJ1 interacts with multiple other pathways to mediate its cytoprotective effects under oxidative stress. These include upstream regulation of AKT, p38 and JNK signaling under stress conditions, mediated via interactions with PTEN, ASK1, and MEKK1 [49]. It is also a known DNA and RNA binding protein with multiple roles in regulating transcription [50], including modulation of chromatin and RNA processing factors [51], [52]. Furthermore, DJ1 levels are found to be reduced in ATM-null cells [53], suggesting coupling to the DNA damage signaling machinery. Notably, DJ1 also enhances transcription of hTERT [54]. Therefore, a role for DJ1 in telomere signaling appears in line with several of these previously reported functions.

The function of PGK1 at telomeres (and those of the other glycolysis pathway components we identified in the TRF2-interactome) is more enigmatic. It has been reported that shelterin component TIN2 also localizes to mitochondria and regulates oxidative phosphorylation [55]. In particular, TIN2 knockdown inhibited glycolysis, leading to increased oxygen consumption and clearly suggesting link between the telosome and cellular metabolism. The current study is in agreement with Lee et al., who previously reported that almost all members of the glycolysis pathway are capable of physical interaction with one or more components of shelterin in the setting of a complementation screen [56]. The glycolytic proteins identified in that study included phosphofructokinase, aldolase, triosephosphate isomerase, glyceraldehyde-3-phosphate dehydrogenase, phosphoglycerate mutase, enolase, and pyruvate kinase, though not PGK1. We found most of these in TRF2 immunoprecipitates. Berthelot et al. also previously identified glyceraldehyde-3-phosphate dehydrogenase as a telomere associated factor using affinity chromatography based on capture by telomeric oligonucleotides [57].

Although glycolysis is traditionally considered to be mainly cytosolic, a number of studies have now demonstrated clearly that multiple glycolytic proteins including PGK1 also exhibit nuclear localization [40], [41]. Hence, the concept of “moonlighting” glycolytic proteins [58] has gained traction. Notably, several glycolytic proteins have also been reported to have specific nuclear functions including DNA binding activities linked to DNA replication/repair and resistance to toxic agents [59], [60], [61]. PGK1 itself was reported some years ago to be a primer recognition protein for DNA polymerase-α with a potential role in lagging-strand synthesis [62]. Since the glycolytic subclusters we identified here are both potentially ATP-generating, an attractive hypothesis is that telomere-association allows for locally high ATP levels through direct recycling of ADP generated during enzymatic remodeling of telomeres and other potentially ATP-intensive processes such as DNA damage signaling.

In a genome-wide siRNA screen for senescence regulators, we recently identified ALDOA as a strong candidate for inhibition to induce accelerated senescence in cancer cells [18]. Our current results shed new light on the complexity of coupling between immortality/senescence and metabolism. We have previously argued that the cell fate switch between immortalisation and senescence exhibits distributed control, sensing inputs from multiple cellular processes [3], [17]. Interactions among telomere components and energy pathways may help explain the known influence of metabolism on cell aging. It is increasingly clear that multiple non-shelterin factors participate in telomere homeostasis [4]. Thus, advanced strategies for manipulating telomere biology either for stability in aging applications or for instability in cancer therapeutics require both more detailed understanding of the telosome and highly selective pharmacological tools.

In summary, from a cell-based screen we have identified a novel pyrazolopyrimidine chemical series capable of modulating metabolic stress-induced telomere homeostasis, of altering the composition of shelterin in unstressed conditions, and which confer protection from certain cytotoxic stressors. A variety of telomere targeting agents have been developed as candidate anti-cancer agents [63], although the telomere protection literature is more sparse. Recently, however, encouraging data have emerged for the Geron Corporation's telomerase activator compound GRN510 [42]. The CRT0063465 mode of action is distinct from previous agents identified in the telomere protection space, being apparently independent of global telomerase levels. Our results suggest that the compound may be a suitable candidate for future interventional studies directed at models of aging or stress responses, either as a single agent or in combination to potentially enhance activity of telomerase-activator or other nuclear remodeling agents such as the recently reported N-acetyltransferase 10 inhibitor Remodelin [64]. In particular, a potentially important area for future investigation could lie in chemoprevention strategies focused on telomeropathy syndromes which produce cancer predisposition.

## Appendix A Supplementary Data

Supplementary File 1: Synthetic routes and data. Image 1

Supplementary File 2: Supplementary figures. Image 2

## References

[1] Lopez-Otin C, Blasco MA, Partridge L, Serrano M, Kroemer G (2013). The hallmarks of aging. Cell.

[2] Bilsland AE, Cairney CJ, Keith WN (2011). Targeting the telomere and shelterin complex for cancer therapy: current views and future perspectives. J Cell Mol Med.

[3] Bilsland AE, Revie J, Keith W (2013). MicroRNA and senescence: the senectome, integration and distributed control. Crit Rev Oncog.

[4] Palm W, de Lange T (2008). How shelterin protects mammalian telomeres. Annu Rev Genet.

[5] Armanios M (2012). Telomerase and idiopathic pulmonary fibrosis. Mutat Res.

[6] Frescas D, de Lange T (2014). A TIN2 dyskeratosis congenita mutation causes telomerase-independent telomere shortening in mice. Genes Dev.

[7] Huang FW, Hodis E, Xu MJ, Kryukov GV, Chin L, Garraway LA (2013). Highly recurrent TERT promoter mutations in human melanoma. Science.

[8] Killedar A, Stutz MD, Sobinoff AP, Tomlinson CG, Bryan TM, Beesley J, Chenevix-Trench G, Reddel RR, Pickett HA (2015). A common cancer risk-associated allele in the hTERT locus encodes a dominant negative inhibitor of telomerase. PLoS Genet.

[9] Mitchell JR, Wood E, Collins K (1999). A telomerase component is defective in the human disease dyskeratosis congenita. Nature.

[10] Vulliamy T, Marrone A, Goldman F, Dearlove A, Bessler M, Mason PJ, Dokal I (2001). The RNA component of telomerase is mutated in autosomal dominant dyskeratosis congenita. Nature.

[11] Saraswat K, Rizvi SI (2017). Novel strategies for anti-aging drug discovery. Expert Opin Drug Discovery.

[12] Ingram DK, Roth GS (2011). Glycolytic inhibition as a strategy for developing calorie restriction mimetics. Exp Gerontol.

[13] Hipkiss AR (2006). Does chronic glycolysis accelerate aging? Could this explain how dietary restriction works? *Annals of the New York Academy of Sciences* **1067**, 361–368. 10.1196/annals.1354.051 16804012

[14] Wardi L, Alaaeddine N, Raad I, Sarkis R, Serhal R, Khalil C, Hilal G (2014). Glucose restriction decreases telomerase activity and enhances its inhibitor response on breast cancer cells: possible extra-telomerase role of BIBR 1532. Cancer Cell Int.

[15] Shaheen F, Grammatopoulos DK, Muller J, Zammit VA, Lehnert H (2014). Extra-nuclear telomerase reverse transcriptase (TERT) regulates glucose transport in skeletal muscle cells. Biochim Biophys Acta.

[16] Bilsland AE, Pugliese A, Liu Y, Revie J, Burns S, McCormick C, Cairney CJ, Bower J, Drysdale M, Narita M (2015). Identification of a selective G1-phase benzimidazolone inhibitor by a senescence-targeted virtual screen using artificial neural networks. Neoplasia.

[17] Bilsland AE, Stevenson K, Liu Y, Hoare S, Cairney CJ, Roffey J, Keith WN (2014). Mathematical model of a telomerase transcriptional regulatory network developed by cell-based screening: analysis of inhibitor effects and telomerase expression mechanisms. PLoS Comput Biol.

[18] Cairney CJ, Godwin LS, Bilsland AE, Burns S, Stevenson KH, McGarry L, Revie J, Moore JD, Wiggins CM, Collinson RS (2017). A 'synthetic-sickness' screen for senescence re-engagement targets in mutant cancer backgrounds. PLoS Genet.

[19] Lafferty-Whyte K, Bilsland A, Hoare SF, Burns S, Zaffaroni N, Cairney CJ, Keith WN (2010). TCEAL7 inhibition of c-Myc activity in alternative lengthening of telomeres regulates hTERT expression. Neoplasia.

[20] Deutsch EW, Csordas A, Sun Z, Jarnuczak A, Perez-Riverol Y, Ternent T, Campbell DS, Bernal-Llinares M, Okuda S, Kawano S (2017). The ProteomeXchange consortium in 2017: supporting the cultural change in proteomics public data deposition. Nucleic Acids Res.

[21] Vizcaino JA, Csordas A, Del-Toro N, Dianes JA, Griss J, Lavidas I, Mayer G, Perez-Riverol Y, Reisinger F, Ternent T (2016). 2016 update of the PRIDE database and its related tools. Nucleic Acids Res.

[22] Gondeau C, Chaloin L, Lallemand P, Roy B, Perigaud C, Barman T, Varga A, Vas M, Lionne C, Arold ST (2008). Molecular basis for the lack of enantioselectivity of human 3-phosphoglycerate kinase. Nucleic Acids Res.

[23] Battye TG, Kontogiannis L, Johnson O, Powell HR, Leslie AG (2011). iMOSFLM: a new graphical interface for diffraction-image processing with MOSFLM. Acta Crystallogr D Biol Crystallogr.

[24] Winn MD, Ballard CC, Cowtan KD, Dodson EJ, Emsley P, Evans PR, Keegan RM, Krissinel EB, Leslie AG, McCoy A (2011). Overview of the CCP4 suite and current developments. Acta Crystallogr D Biol Crystallogr.

[25] McCoy AJ, Grosse-Kunstleve RW, Adams PD, Winn MD, Storoni LC, Read RJ (2007). Phaser crystallographic software. J Appl Cryst.

[26] Emsley P, Lohkamp B, Scott WG, Cowtan K (2010). Features and development of Coot. Acta Crystallogr D Biol Crystallogr.

[27] Murshudov GN, Vagin AA, Lebedev A, Wilson KS, Dodson EJ (1999). Efficient anisotropic refinement of macromolecular structures using FFT. Acta Crystallogr D Biol Crystallogr.

[28] Xing L, Klug-Mcleod J, Rai B, Lunney EA (2015). Kinase hinge binding scaffolds and their hydrogen bond patterns. Bioorg Med Chem.

[29] Sanchez-Arago M, Cuezva JM (2011). The bioenergetic signature of isogenic colon cancer cells predicts the cell death response to treatment with 3-bromopyruvate, iodoacetate or 5-fluorouracil. J Transl Med.

[30] Wilson MA, Collins JL, Hod Y, Ringe D, Petsko GA, National Academy of Sciences of the United States of America (2003). The 1.1-A resolution crystal structure of DJ-1, the protein mutated in autosomal recessive early onset Parkinson's disease *Proceedings of the*.

[31] Canet-Aviles RM, Wilson MA, Miller DW, Ahmad R, McLendon C, Bandyopadhyay S, Baptista MJ, Ringe D, Petsko GA, Cookson MR (2004). The Parkinson's disease protein DJ-1 is neuroprotective due to cysteine-sulfinic acid-driven mitochondrial localization. Proc Natl Acad Sci U S A.

[32] Kato I, Maita H, Takahashi-Niki K, Saito Y, Noguchi N, Iguchi-Ariga SM, Ariga H (2013). Oxidized DJ-1 inhibits p53 by sequestering p53 from promoters in a DNA-binding affinity-dependent manner. Mol Cell Biol.

[33] Landon MR, Lieberman RL, Hoang QQ, Ju S, Caaveiro JM, Orwig SD, Kozakov D, Brenke R, Chuang GY, Beglov D (2009). Detection of ligand binding hot spots on protein surfaces via fragment-based methods: application to DJ-1 and glucocerebrosidase. J Comput Aided Mol Des.

[34] Choi D, Kim J, Ha S, Kwon K, Kim EH, Lee HY, Ryu KS, Park C (2014). Stereospecific mechanism of DJ-1 glyoxalases inferred from their hemithioacetal-containing crystal structures. FEBS J.

[35] Lee JY, Song J, Kwon K, Jang S, Kim C, Baek K, Kim J, Park C (2012). Human DJ-1 and its homologs are novel glyoxalases. Hum Mol Genet.

[36] Mihoub M, Abdallah J, Richarme G (2017). Protein repair from glycation by glyoxals by the DJ-1 family maillard deglycases. Adv Exp Med Biol.

[37] Richarme G, Liu C, Mihoub M, Abdallah J, Leger T, Joly N, Liebart JC, Jurkunas UV, Nadal M, Bouloc P (2017). Guanine glycation repair by DJ-1/Park7 and its bacterial homologs. Science.

[38] Knobbe CB, Revett TJ, Bai Y, Chow V, Jeon AH, Bohm C, Ehsani S, Kislinger T, Mount HT, Mak TW (2011). Choice of biological source material supersedes oxidative stress in its influence on DJ-1 in vivo interactions with Hsp90. J Proteome Res.

[39] Haas J, Roth S, Arnold K, Kiefer F, Schmidt T, Bordoli L, Schwede T (2013). The Protein Model Portal--a comprehensive resource for protein structure and model information *Database : the journal of biological databases and curation* **2013**, bat031. 10.1093/database/bat031 PMC3889916 23624946

[40] Qattan AT, Radulovic M, Crawford M, Godovac-Zimmermann J (2012). Spatial distribution of cellular function: the partitioning of proteins between mitochondria and the nucleus in MCF7 breast cancer cells. J Proteome Res.

[41] Shakib K, Norman JT, Fine LG, Brown LR, Godovac-Zimmermann J (2005). Proteomics profiling of nuclear proteins for kidney fibroblasts suggests hypoxia, meiosis, and cancer may meet in the nucleus. Proteomics.

[42] Le Saux CJ, Davy P, Brampton C, Ahuja SS, Fauce S, Shivshankar P, Nguyen H, Ramaseshan M, Tressler R, Pirot Z (2013). A novel telomerase activator suppresses lung damage in a murine model of idiopathic pulmonary fibrosis. PloS one.

[43] Ke N, Wang X, Xu X, Abassi YA (2011). The xCELLigence system for real-time and label-free monitoring of cell viability. Methods Mol Biol.

[44] Dongiovanni P, Valenti L, Ludovica Fracanzani A, Gatti S, Cairo G, Fargion S (2008). Iron depletion by deferoxamine up-regulates glucose uptake and insulin signaling in hepatoma cells and in rat liver. Am J Pathol.

[45] Dayani PN, Bishop MC, Black K, Zeltzer PM (2004). Desferoxamine (DFO)–mediated iron chelation: rationale for a novel approach to therapy for brain cancer. J Neurooncol.

[46] So EY, Ausman M, Saeki T, Ouchi T (2011). Phosphorylation of SMC1 by ATR is required for desferrioxamine (DFO)-induced apoptosis. Cell Death Dis.

[47] Fortin PM, Fisher SA, Madgwick KV, Trivella M, Hopewell S, Doree C, Estcourt LJ (2018). Interventions for improving adherence to iron chelation therapy in people with sickle cell disease or thalassaemia *The Cochrane database of systematic reviews* **5**, CD012349. 10.1002/14651858.CD012349.pub2 PMC5985157 29737522

[48] Requejo-Aguilar R, Lopez-Fabuel I, Jimenez-Blasco D, Fernandez E, Almeida A, Bolanos JP (2015). DJ1 represses glycolysis and cell proliferation by transcriptionally up-regulating Pink1. Biochem J.

[49] Cao J, Lou S, Ying M, Yang B (2015). DJ-1 as a human oncogene and potential therapeutic target. Biochem Pharmacol.

[50] Biosa A, Sandrelli F, Beltramini M, Greggio E, Bubacco L, Bisaglia M (2017). Recent findings on the physiological function of DJ-1: Beyond Parkinson's disease. Neurobiol Dis.

[51] Zhong N, Kim CY, Rizzu P, Geula C, Porter DR, Pothos EN, Squitieri F, Heutink P, Xu J (2006). DJ-1 transcriptionally up-regulates the human tyrosine hydroxylase by inhibiting the sumoylation of pyrimidine tract-binding protein-associated splicing factor. J Biol Chem.

[52] Xu J, Zhong N, Wang H, Elias JE, Kim CY, Woldman I, Pifl C, Gygi SP, Geula C, Yankner BA (2005). The Parkinson's disease-associated DJ-1 protein is a transcriptional co-activator that protects against neuronal apoptosis. Hum Mol Genet.

[53] Valentin-Vega YA, Maclean KH, Tait-Mulder J, Milasta S, Steeves M, Dorsey FC, Cleveland JL, Green DR, Kastan MB (2012). Mitochondrial dysfunction in ataxia-telangiectasia. Blood.

[54] Sitaram RT, Cairney CJ, Grabowski P, Keith WN, Hallberg B, Ljungberg B, Roos G (2009). The PTEN regulator DJ-1 is associated with hTERT expression in clear cell renal cell carcinoma. International journal of cancer Journal international du cancer.

[55] Chen LY, Zhang Y, Zhang Q, Li H, Luo Z, Fang H, Kim SH, Qin L, Yotnda P, Xu J (2012). Mitochondrial localization of telomeric protein TIN2 links telomere regulation to metabolic control. Mol Cell.

[56] Lee OH, Kim H, He Q, Baek HJ, Yang D, Chen LY, Liang J, Chae HK, Safari A, Liu D, et al. (2011). Genome-wide YFP fluorescence complementation screen identifies new regulators for telomere signaling in human cells *Molecular & cellular proteomics : MCP* 10, M110 001628. 10.1074/mcp.M110.001628 PMC3033672 21044950

[57] Berthelot V, Mouta-Cardoso G, Hegarat N, Guillonneau F, Francois JC, Giovannangeli C, Praseuth D, Rusconi F (2016). The human DNA ends proteome uncovers an unexpected entanglement of functional pathways. Nucleic Acids Res.

[58] Erez A, DeBerardinis RJ (2015). Metabolic dysregulation in monogenic disorders and cancer - finding method in madness. Nat Rev Cancer.

[59] Ronai Z (1993). Glycolytic enzymes as DNA binding proteins. Int J Biochem.

[60] Satou W, Tanimoto H, Ukekawa R, Fujii M, Ayusawa D (2004). Amplification of nuclear aldolase A in mouse cell mutants resistant to Hoechst 33342. Biochem Biophys Res Commun.

[61] Wang W, Wang L, Endoh A, Hummelke G, Hawks CL, Hornsby PJ (2005). Identification of alpha-enolase as a nuclear DNA-binding protein in the zona fasciculata but not the zona reticularis of the human adrenal cortex. J Endocrinol.

[62] Kumble KD, Iversen PL, Vishwanatha JK (1992). The role of primer recognition proteins in DNA replication: inhibition of cellular proliferation by antisense oligodeoxyribonucleotides. J Cell Sci.

[63] Neidle S (2016). Quadruplex nucleic acids as novel therapeutic targets. J Med Chem.

[64] Balmus G, Larrieu D, Barros AC, Collins C, Abrudan M, Demir M, Geisler NJ, Lelliott CJ, White JK, Karp NA (2018). Targeting of NAT10 enhances healthspan in a mouse model of human accelerated aging syndrome. Nat Commun.

