# Supplementary material for: A Novel Pyrazolopyrimidine Ligand of Human PGK1 and Stress Sensor DJ1 Modulates the Shelterin Complex and Telomere Length Regulation
Source: Neoplasia. 2019 Aug 8;21(9):893–907. doi: 10.1016/j.neo.2019.07.008 (PMC6700475; doi:10.1016/j.neo.2019.07.008)
Supplement: Supplementary file 1 — Synthetic routes and data. [file mmc1.docx]

**SUPPLEMENTAL FILE 1: CHEMICAL SYNTHESES ROUTES AND DATA**

***Nuclear Magnetic Resonance (NMR) and High Performance Liquid Chromatography-Mass Spectrometry (HPLC-MS)***

Proton NMR spectra were recorded using a Bruker AMX-300 at 300 MHz. Shifts were reported in ppm relative to tetramethylsilane internal standard or residual protic solvent. HPLC columns were 5 μm pore size, C18 column (50 x 4.60 mm). Two HPLC-MS methods were used. Method 1 employed Gilson (Bedfordshire, UK) 306 pumps, 811C mixer, 806 manometric module, and UV/VIS 152 detector at 254 nm wavelength. MS was performed using 10 μl injection volume, Finnigan AQA with Waters SunFire. Water and acetonitrile mobile phase contained 0.1% formic acid. Eluent flow rate was 1.5 mL/min, using linear 95% water: 5% acetonitrile/ 5% water: 95% acetonitrile gradient over 5.5 minutes, then maintained for 2 minutes.

Method 2 employed an Alliance e2695 with a Waters 2998 diode array (210-600 nm) detector operated at 254 nm. MS was performed with Acquity SQ, detecting masses between 100 and 700 g/mol. Injection volume was 10 µL at a maximum concentration of 1 mg/mL. Flow rate was 1.5 mL/min. Mobile phases and elution conditions were as above, with 10 minute gradient and final conditions held for 0.5 min before returning eluent level to starting conditions over 6 seconds. Column equilibration was performed before each injection.

***Synthesis of 3-[2-(4-bromophenyl)-5,7-dimethyl-pyrazolo[1,5-a]pyrimidin-6-yl]propanoic acid (CRT0063465)***

*Step 1: Methyl 3-[2-(4-bromophenyl)-5,7-dimethyl-pyrazolo[1,5-a]pyrimidin-6-yl]propanoate*

5-amino-3-(4-bromophenyl)pyrazole (120 mg, 0.5 mmol), methyl 4-acetyl-5-oxo hexanoate (0.087 mL, 0.5 mmol) and acetic acid (1 mL) were stirred at 100° C for 1 h. The reaction was cooled, 1 mL water added, and stirred at room temperature overnight. Off white solid was collected by filtration, washed with cold MeOH and dried, yielding methyl 3-[2-(4-bromophenyl)-5,7-dimethyl-pyrazolo[1,5-a]pyrimidin-6-yl]propanoate (150 mg, 79 % yield). LCMS: RT 6.35 min, MI 390.2, Method 1; ^1^H NMR (300 MHz, CDCl_3_) δ 7.92 (2H, d *J* = 9 Hz), 7.62 (2H, d *J* = 9 Hz), 6.79 (1H, s), 3.76 (3H, s), 3.09 (2H, m), 2.84 (3H, s), 2.56 (3H, s), 2.51 (2h, m).

*Step 2: 3-[2-(4-bromophenyl)-5,7-dimethyl-pyrazolo[1,5-a]pyrimidin-6-yl]propanoic acid*

Methyl 3-[2-(4-bromophenyl)-5,7-dimethyl-pyrazolo[1,5-a]pyrimidin-6-yl]propanoate (60 mg, 0.2 mmol), EtOH (3 mL), and 2N KOH (0.4 mmol, 0.2 mL) were heated at reflux for 2 h. The reaction was cooled then evaporated under reduced pressure to give the title compound as colourless solid (40 mg, 80 % yield as the potassium salt). LCMS: RT 6.04 min, MI 374.11, Method 2; ^1^H NMR (300 MHz, D_2_O) δ 7.98 (2H, d *J* = 9 Hz), 7.67 (2H, d *J* = 9 Hz), 7.00 (1H, s), 2.79 (2H, m), 2.53 (3H, s), 2.49 (3H, s), 1.99 (2h, m).

***Synthesis of 3[2-(4-Azido-phenyl)-5,7-dimethylpyrazolo[1,5-a]pyrimidin-6-yl]-propionic acid methyl ester (CRT0066127)***

*Step 1: Methyl 3-[5,7-dimethyl-2-(4-nitrophenyl)pyrazolo[1,5-a]pyrimidin-6-yl]propanoate*

5-amino-3-(4-nitrophenyl)pyrazole (690 mg, 3.4 mmol), methyl 4-acetyl-5-oxo hexanoate (0.59 mL, 3.4 mmol), and acetic acid (10 mL) were stirred at 100° C for 1 h. The reaction was cooled, 10 mL water added, and stirred at room temperature overnight. Off-white solid was collected by filtration, washed with cold MeOH, then dried, giving methyl 3-[5,7-dimethyl-2-(4-nitrophenyl)pyrazolo[1,5-a]pyrimidin-6-yl]propanoate as off-white solid (750 mg, 62 % yield), used in the next step without further purification. LCMS: RT 7.5 min, MI 355, Method 2.

*Step 2: Methyl 3-[2-(4-aminophenyl)-5,7-dimethyl-pyrazolo[1,5-a]pyrimidin-6-yl]propanoate*

Methyl 3-[5,7-dimethyl-2-(4-nitrophenyl)pyrazolo[1,5-a]pyrimidin-6-yl]propanoate (0.25 g, 0.7 mmol), EtOH (5 mL), cyclohexene (1.4 mL, 14 mmol) and 10 % Pd/C (10 mg) were heated in a microwave at (140 °C, 10 minutes). The reaction was cooled, filtered through a PTFE frit, and evaporated under reduced pressure, giving methyl 3-[2-(4-aminophenyl)-5,7-dimethyl-pyrazolo[1,5-a]pyrimidin-6-yl]propanoate as pale yellow oil, 0.33g (> 100 % yield), used in the next step without further purification. LCMS: RT 4.1 min, MI (MH+) 325, Method 2.

*Step 3: 3-[2-(4-amino-3,5-dibromo-phenyl)-5,7-dimethyl-pyrazolo[1,5a]pyrimidin-6-yl]-propionic acid methyl ester*

Methyl 3-[2-(4-aminophenyl)-5,7-dimethyl-pyrazolo[1,5-a]pyrimidin-6-yl]propanoate (0.10 g, 0.32 mmol), DCM (4 mL), and sodium acetate (84 mg, 1.02 mmol) were stirred at room temperature. Pyridinium tribromide (239 mg, 0.67 mmol) was added portion wise. The reaction turned from pale straw colour to an orange suspension. After stirring at room temperature for 3 hours, 0.1 M sodium metabisulfite (pH 4, 10 mL) was added. The organic layer was extracted, washed with water, dried (MgSO4), filtered and evaporated under reduced pressure, giving 3-[2-(4-amino-3,5-dibromo-phenyl)-5,7-dimethyl-pyrazolo[1,5a]pyrimidin-6-yl]-propionic acid methyl ester (140 mg, 91 % yield), used in the next step without further purification. LCMS: RT 7.8 min, MI (MH+) 483.2, Method 2.

*Step 4: 3[2-(4-Amino-phenyl)-5,7-dimethylpyrazolo[1,5-a]pyrimidin-6-yl]-propionic acid methyl ester*

3-[2-(4-Amino-3,5-dibromo-phenyl)-5,7-dimethyl-pyrazolo[1,5a]pyrimidin-6-yl]-propionic acid methyl ester (50mg, 0.104mmol), 10% Pd/C (5 mg, 10wt%) and sodium acetate (35mg, 0.427 mmol, 4.1 equivalents) were charged to a flask with ethyl acetate (5 ml). The mixture was evacuated, backfilled with nitrogen three times, then placed under vacuum. Stirring was performed under hydrogen atmosphere at room temperature for 19 hours. Analysis at this time by LCMS showed complete conversion. The reaction was evacuated and backfilled with nitrogen three times. Catalyst was removed by filtration through Celite©, and the Celite© washed with ethyl acetate. The ethyl acetate was washed with 2x10 ml water (both pH4) and 2 x 10 ml saturated sodium hydrogen carbonate (both pH 8-9). The organic layer was dried over magnesium sulfate, filtered and concentrated under reduced pressure, giving 3[2-(4-Amino-phenyl)-5,7-dimethylpyrazolo[1,5-a]pyrimidin-6-yl]-propionic acid methyl ester, 28 mg (82%), used in the next step without further purification. LCMS: RT 4.0 min, MI (MH+) 325, Method 2; TLC (silica, 254nm) using 10% Ethyl acetate in DCM as eluent showed the main band at Rf 0.2, with a faint shadow at Rf 0.1.

*Step 5: 3-[2-(4-azido-phenyl)-5,7-dimethyl-pyrazolo[1,5a]pyrimidin-6-yl]-propionic acid methyl ester (CRT0066127)*

3 -(2- (4-Amino-phenyl)- 5,7- dimethyl- pyrazolo[1,5a]pyrimidin-6-yl]-propionic acid methyl ester (50 mg, 0.154 mmol) was dissolved in 1M HCl (2 ml) and cooled in an ice-salt bath. Sodium nitrite (16 mg, 0.231 mmol, 1.5 equivalents) was dissolved in 1 ml water and added dropwise via Pastuer pipette to the reaction over a period of 5 minutes. The mixture was stirred in the cooling bath for 1 hour. Meanwhile, sodium acetate (101 mg, 1.23 mmol, 8.0 equivalents) and sodium azide (15 mg, 0.231 mmol, 1.5 equivalents) were combined, dissolved in 1ml water and added to the reaction over a period of 10 minutes. During this addition, thick cream precipitate formed. The reaction was stirred for one hour in the cooling bath, then for one hour at room temperature, at which time LCMS showed complete conversion to product. DCM (10 ml) was added and the reaction adjusted to pH 14 with 6 M NaOH (0.6 ml). The layers were separated and the aqueous layer extracted with further DCM (5 ml). The organic layers were combined, dried over magnesium sulfate and concentrated under reduced pressure to give the final product 3-[2-(4-azido-phenyl)-5,7-dimethyl-pyrazolo[1,5a]pyrimidin-6-yl]-propionic acid methyl ester (CRT0066127, 50 mg, 93%) as a pale yellow solid. LCMS: RT 9.3 min, MI (MH+) 337, Method 1; ^1^H NMR (300 MHz, CDCl_3_) δ 8.42 (1H, s), 8.13 (2H, d *J* = 9 Hz), 7.44 (2H, d *J* = 9 Hz), 3.72 (3H, s), 3.09 (2H, m), 2.75 (3H, s), 2.67 (3H, s), 2.53 (2h, m); TLC (silica, 254 nm) using neat DCM as eluent shows main band at Rf 0.3.

***Synthesis of 3-[2-(4-azido-3,5-^3^H_2_-phenyl)-5,7-dimethyl-pyrazolo[1,5a]pyrimidin-6-yl]-propionic acid methyl ester (CRT0105481)***

Tritiated derivative CRT0105481 was synthesised using the above scheme at Selcia (Tritec). Radio-purity was > 96%, with specific activity 2 mCi/mol. The compound was stored in ethanol solution in the dark at 1 mCi.
