# Supplementary material for: A Novel Pyrazolopyrimidine Ligand of Human PGK1 and Stress Sensor DJ1 Modulates the Shelterin Complex and Telomere Length Regulation
Source: Neoplasia. 2019 Aug 8;21(9):893–907. doi: 10.1016/j.neo.2019.07.008 (PMC6700475; doi:10.1016/j.neo.2019.07.008)
Supplement: Supplementary file 2 — Supplementary figures. [file mmc2.docx]

**SUPPLEMENTAL FILE 2: SUPPORTING FIGURES**

**
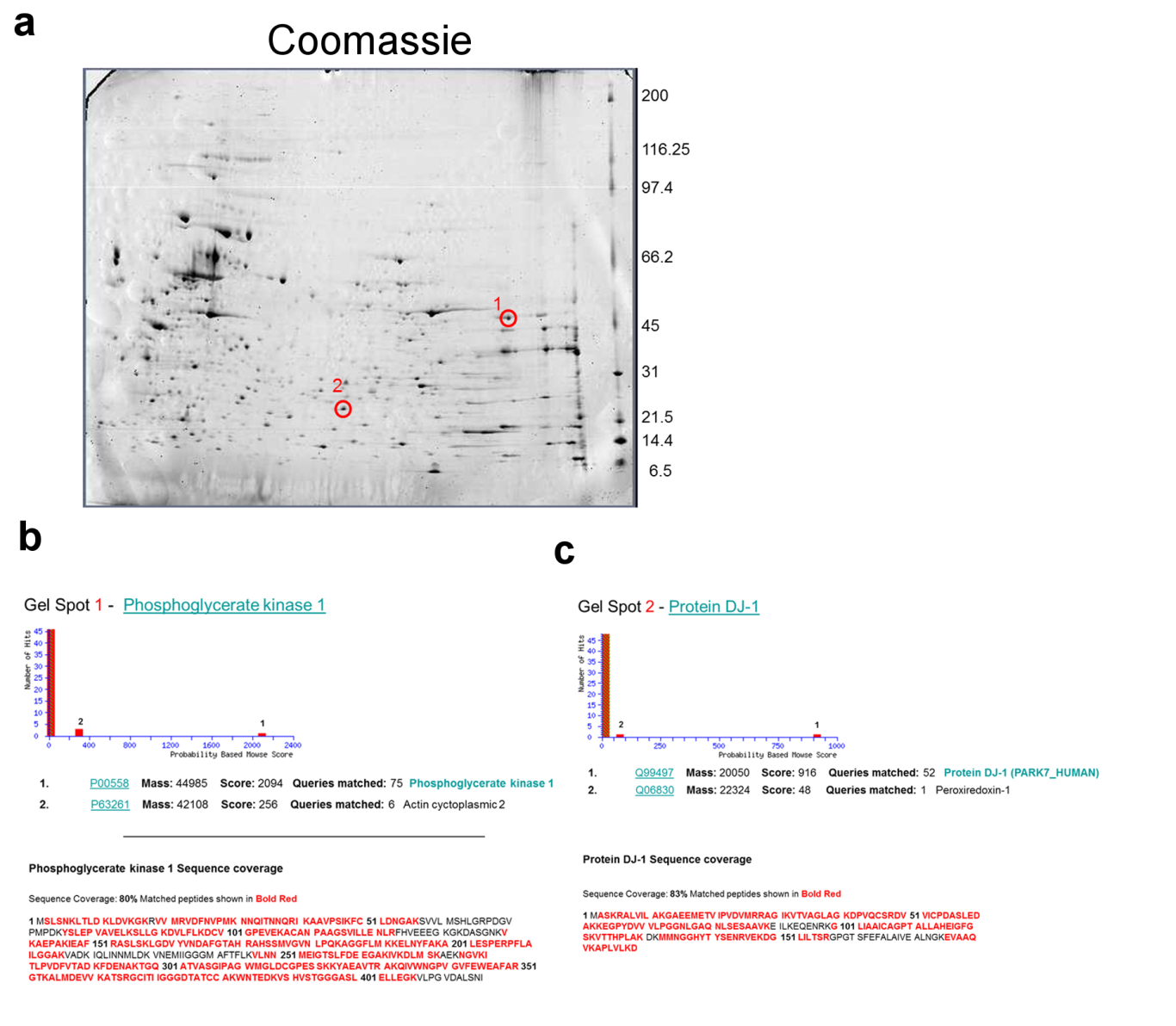
**

**Figure S1: Identification of PGK1 and DJ1 as targets of photoaffinity probe CRT0105481.** A2780 cells were incubated for 30 minutes in 10 nM or 100 nM tritiated CRT0105481 then exposed to 120 mJ 254 nm UV light using a Stratalinker and harvested for 2D gel analysis and autoradiography. Gels were stained, infused with En3hance autoradiography enhancer reagent, dried, and incubated with radiographic film for 1 week (100 nM) or 1 month (10 nM) at -70 ^o^C. Labelled spots were excised for analysis by LC-MS/MS. (a) Stained 2D gel spots on the 10 nM gel. The spots circled in red were found to be labelled by the photoaffinity compound. (b) MS identification of gel spot 1 as PGK1. (c) MS identification of gel spot 2 as DJ1. The same results were obtained at 100 nM compound.


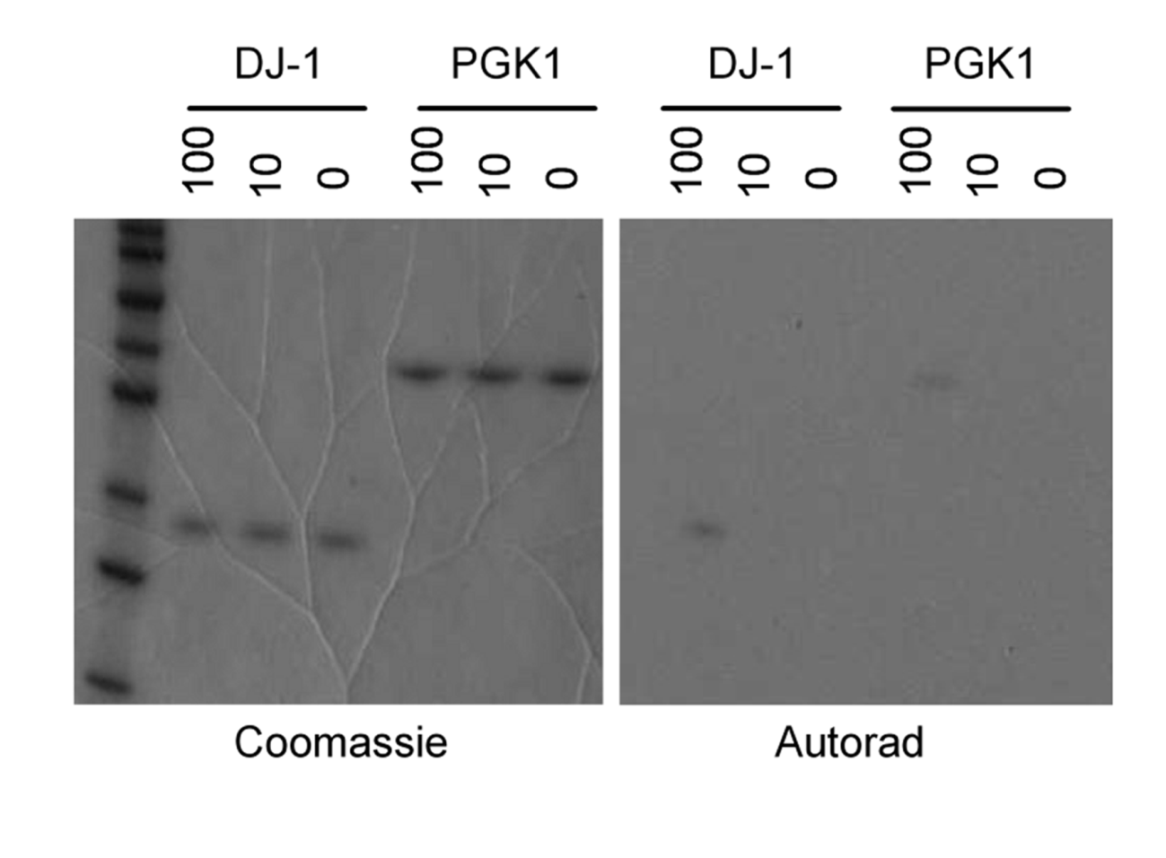


**Figure S2: CRT0105481 binds to PGK1 and DJ1 in vitro.** 500 ng of recombinant human PGK1 (Ab211320, Abcam, UK) or DJ1 (Ab51198, Abcam, UK) were incubated alone or in the presence of 10 nM or 100 nM CRT0105481 for 5 minutes then binding reactions exposed to 120 mJ 254 nm UV light using a Stratalinker (Agilent, Santa Clara, CA). Proteins were separated by SDS-PAGE and gels were stained with simply blue, dried, and incubated with autoradiography film for 5 days. Labelled bands were detected for both proteins at 100 nM compound after this period. Note that, although no signal was observed at 10 nM, in our original in-cell labelling experiments using 10 nM (figure S1), gels were incubated with radiographic film for one month prior to exposure due to the 10-fold reduction in 3H relative to 100 nM. The signal observed with 5 day incubation in this experiment is consistent with in-cell labelling observed at 100 nM after 1 week incubation.


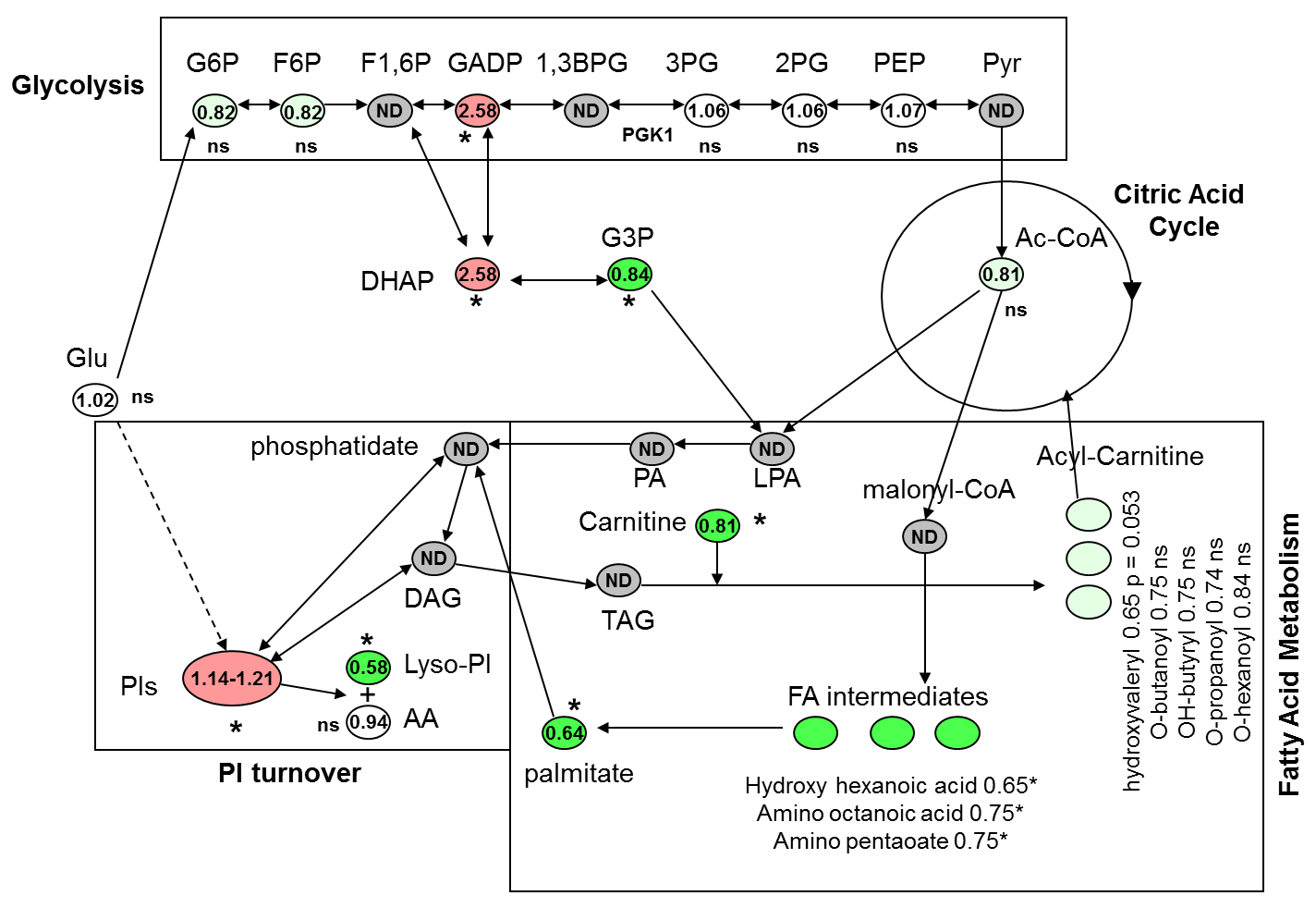


**Figure S3: HCT116 cell metabolite network affected by CRT0063465 treatment.** HCT116 cells were treated for 2 h in 6 well plates with 10 nM CRT0063465 or vehicle. Supernatants were then removed and cells were harvested by scraping in 400µL per well of Chloroform/Methanol/Water (1:3:1 ratio) at 4^o^C. Cell suspensions were then placed on a rocker at 4^o^C for 1 hour then vortex at 4^o^C for 5 minutes. Finally, cells were pelleted by centrifugation for 3 minutes at 13,000g at 4^o^C and supernatants stored at -80o C until analysis by LC–MS. The metabolomics analysis was performed by Glasgow Polyomics (<http://www.polyomics.gla.ac.uk/>). Identified masses were mapped against a database of 41623 search masses for metabolite-calling. The experiments were performed three times. Further information is given in supporting S1 Table.

**
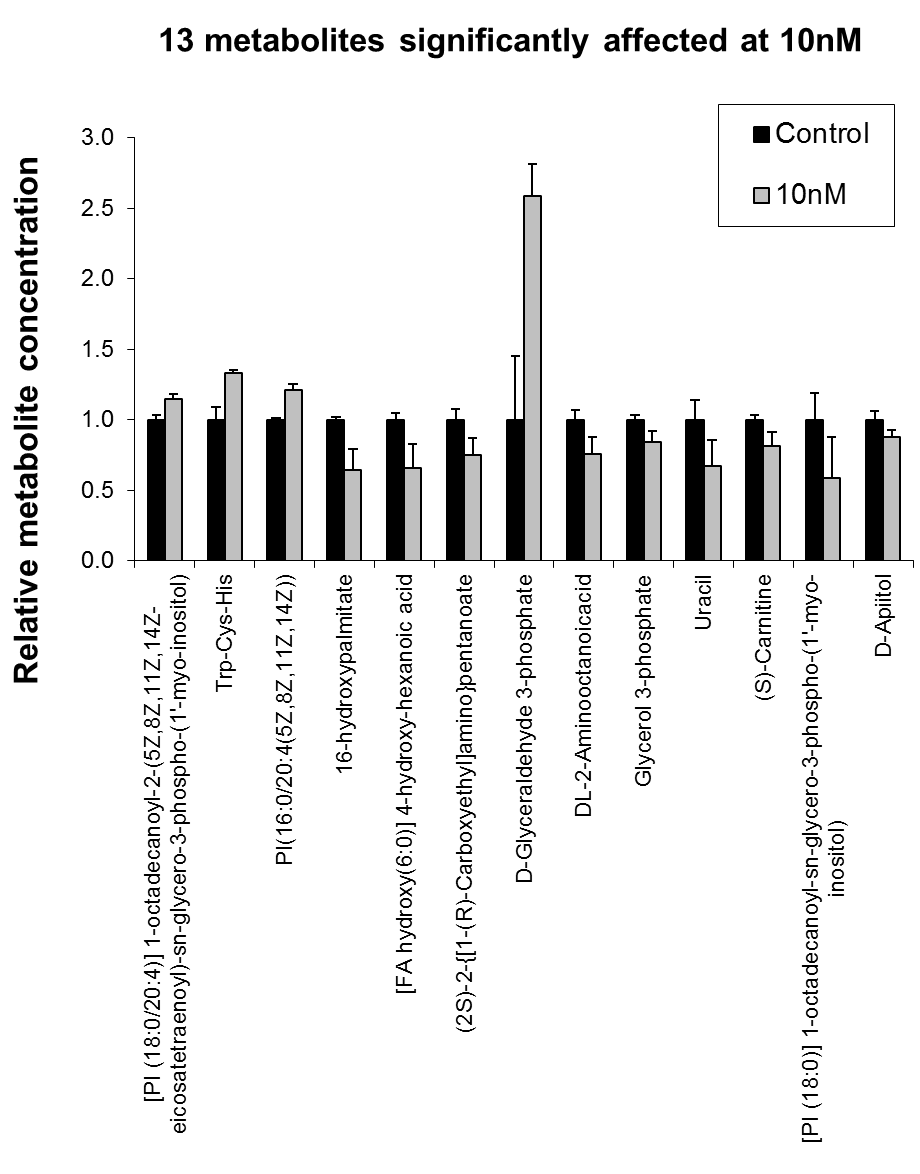
**

**Figure S4: All putative metabolites significantly affected by CRT0063465 treatment.** Cell treatments, harvesting and analysis were performed as described in the legend to figure S3. 13 entities were significantly affected (p<0.05 by t-test). Values shown are mean ± SD of 3 experiments normalised to control (vehicle treatment) for each metabolite called. Further information on each call is given in supporting S1 Table below.

|  | | | |  | | |
| --- | --- | --- | --- | --- | --- | --- |
| CRT No | R_1_ | R_2_ | hTR IC_50_ | CRT No | R_3_ | hTR IC_50_ |
| CRT0063463 |  | H | < 10 nM | CRT0063459 |  | < 1 nM |
| CRT0063465 |  | H | < 1 nM | CRT0063465 |  | < 1 nM |
| CRT0098829 |  | H | 260 nM | CRT0064499 |  | > 10 μM |
| CRT0063466 |  | H | < 1 nM | CRT0098921 |  | 4 μM |
| CRT0063468 |  | H | > 10 μM | CRT0098917 |  | > 10 μM |
| CRT0066127 |  | H | < 10 nM | CRT0098925 |  | > 10 μM |
| CRT0098922 | H |  | > 10 μM | CRT0098923 | H | > 10 μM |

**Figure S5: Key structure-activity relationships around the series scaffold in the A2780 cell-based telomerase promoter assay.** The CRT0063465 bromoaryl ring and related substituents at R1 bind the PGK1 nucleotide binding pocket, while substituent R3 is proposed to interact with DJ1. The CRT0063465 carboxylate acid group is negatively charged at physiological pH, allowing electrostatic interaction with oxidised DJ1 C106.  The active ester compound CRT0063459 is expected to be hydrolysed to the acid inside the cell. Experiments with several other acid/ester matched pairs indicated equipotent activity (not shown).  Compounds CRT0098923/ CRT0098925 do not contain any hydrogen bonding capacity, while CRT0098921 can act as a hydrogen bond acceptor/ donor but is not negatively charged, as is also the case for the carboxamide CRT0064499.  Interestingly, CRT0098917 is an ester which may be expected to be hydrolysed to an acid in the cell. However, due to the shorter side chain, the ester is flanked in this case by two adjacent methyl groups. This would cause considerable steric hindrance to prevent or slow hydrolysis.  Our own attempt to convert the CRT0098917 to an acid under forcing conditions produced no product. The hTR assay is described in Pubchem AID 1259345.


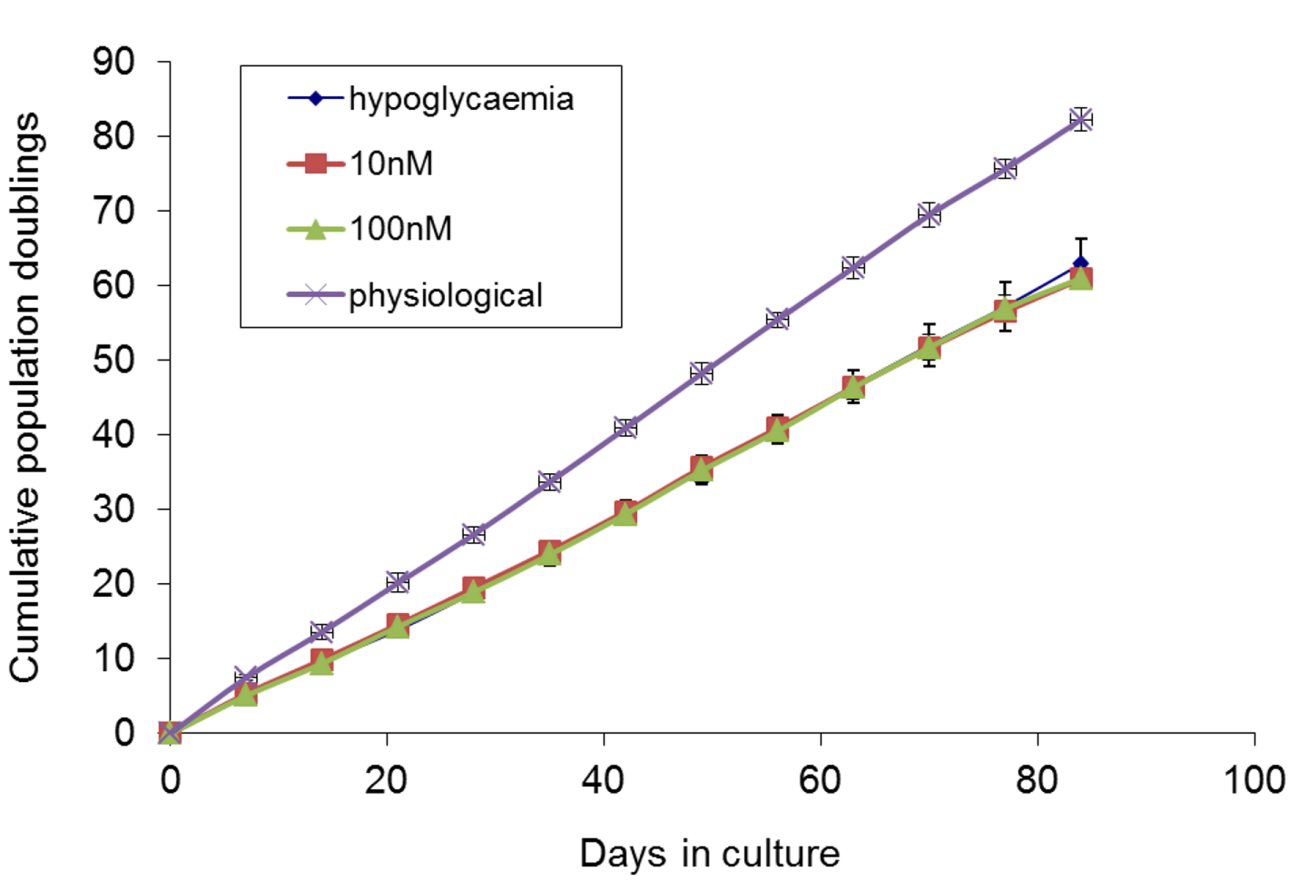


**Figure S6: Cell growth kinetics under physiological or hypoglycaemic culture conditions.** HCT116 colorectal cancer cells were maintained in long term culture with twice-weekly feeding using physiological (5 mM) or hypoglycemic (500 μM) glucose conditions for 84 days. Cells were counted weekly for evaluation of cumulative population doublings and samples were harvested weekly for TRAP and telomere length analysis (main text). Cells grown under hypoglycaemia grew stably in long term culture, though at reduced rate. Addition of 10 nM or 100 nM CRT0063465 to the hypoglycemic conditions had no combinatorial effect on cell growth. The experiment was performed 3 times with similar results. Mean ± SEM of 3 experiments is shown.

**
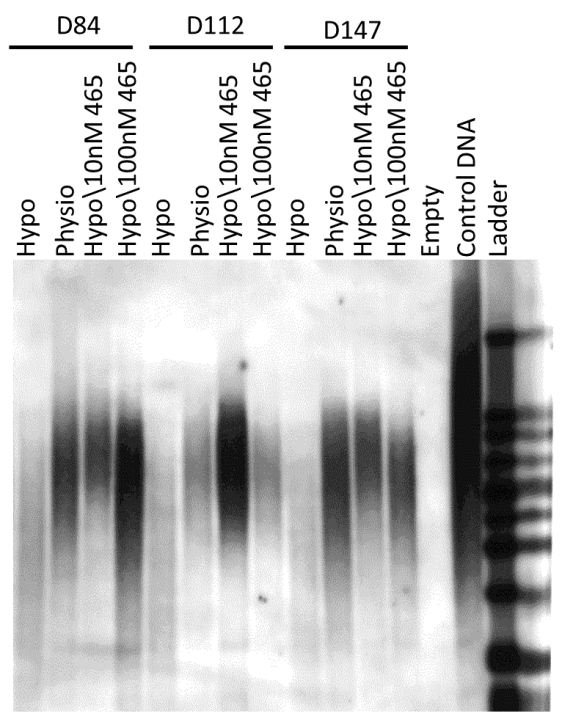
**

**Figure S7: Long term protection from telomere shortening in hypoglycaemia by CRT0063465**. In one experiment, HCT116 cells were cultured for 147 days in medium containing 5 mM (physiological) or 500 μM (hypoglycaemic) glucose in the presence of absence of 10 nM or 100 nM CRT0063465. Samples were taken weekly. Late time point samples from this experiment were analysed for telomere length by Telomere Restriction Fragment (TRF)-Southern blotting. Results remained consistent with those reported up to day 84 in the text.

**
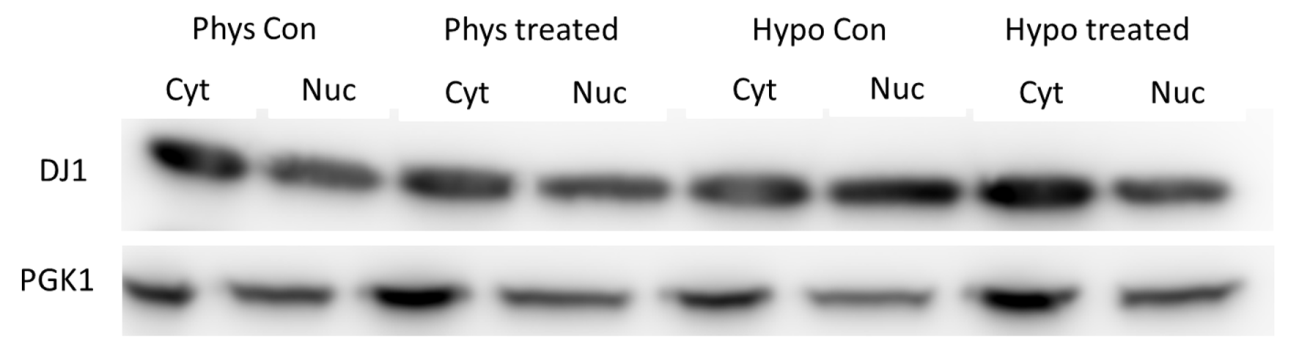
**

**Figure S8: PGK1 and DJ1 are present in both cytoplasm and nucleus**. HCT116 cells were grown for 1 week in the presence of physiological glucose or hypoglycaemia (500 μM glucose) with or without 10 nM CRT0063465. After 1 week treatment, cells were harvested and nuclear and cytoplasmic extracts prepared for western blotting analysis of PGK1 and DJ1 levels as described in online methods. Both proteins were found to be localised in both compartments. Hypoglycaemia and compound treatments had little effect on localisation of either protein.

| Mass | RT | Formula | Isomers | Metabolite call | Confidence | P-value |
| --- | --- | --- | --- | --- | --- | --- |
| 886.56 | 3.551 | C47H83O13P | 19 | [PI (18:0/20:4)] 1-octadecanoyl-2-(5Z,8Z,11Z,14Z-eicosatetraenoyl)-sn-glycero-3-phospho-(1'-myo-inositol) | 5 | 0.013 |
| 444.16 | 10.966 | C20H24N6O4S | 1 | Trp-Cys-His | 5 | 0.015 |
| 858.53 | 3.555 | C45H79O13P | 15 | PI(16:0/20:4(5Z,8Z,11Z,14Z)) | 5 | 0.018 |
| 272.24 | 3.662 | C16H32O3 | 18 | 16-hydroxypalmitate | 7 | 0.019 |
| 132.08 | 4.755 | C6H12O3 | 14 | [FA hydroxy(6:0)] 4-hydroxy-hexanoic acid | 5 | 0.022 |
| 189.10 | 7.250 | C8H15NO4 | 5 | (2S)-2-{[1-(R)-Carboxyethyl]amino}pentanoate | 7 | 0.022 |
| 170.00 | 15.166 | C3H7O6P | 7 | D-Glyceraldehyde 3-phosphate | 8 | 0.024 |
| 159.13 | 12.788 | C8H17NO2 | 4 | DL-2-Aminooctanoicacid | 5 | 0.026 |
| 172.01 | 13.972 | C3H9O6P | 3 | Glycerol 3-phosphate | 5 | 0.036 |
| 112.03 | 8.365 | C4H4N2O2 | 2 | Uracil | 10 | 0.037 |
| 161.11 | 12.786 | C7H15NO3 | 2 | (S)-Carnitine | 7 | 0.045 |
| 600.33 | 4.022 | C27H53O12P | 1 | [PI (18:0)] 1-octadecanoyl-sn-glycero-3-phospho-(1'-myo-inositol) | 5 | 0.047 |
| 136.07 | 9.834 | C5H12O4 | 2 | D-Apiitol | 7 | 0.047 |

Table S1: Data for significant entities in metabolomics analysis. Confidence of 10 corresponds to an internal standard included in the set of runs.
